# Cardiac microphysiological systems in cardiovascular research: Construction paradigms, maturation trajectories, and translational frontiers

**DOI:** 10.1002/btm2.70167

**Published:** 2026-08-09

**Authors:** Rongfang Xie, Haiyang Zhao, Yuqing Lei, Xinrui Wang

**Affiliations:** ^1^ Medical Research Center, Fujian Maternity and Child Health Hospital, College of Clinical Medicine for Obstetrics & Gynecology and Pediatrics, Fujian Medical University Fuzhou China; ^2^ Fujian Key Laboratory of Women and Children's Critical Diseases Research, Fujian Maternity and Child Health Hospital Fuzhou China; ^3^ School of Medicine, Fuzhou University Fuzhou China

**Keywords:** cardiac microphysiological systems, cardiac organoids, disease modeling, engineered cardiac tissue models, maturation assessment

## Abstract

Cardiovascular diseases remain the leading cause of global mortality, yet traditional preclinical models fail to accurately capture the physiological and genetic complexity of the human heart, hindering the development of targeted therapies. Cardiac microphysiological systems (cardiac MPS), including self‐organizing human cardiac organoids and engineered cardiac tissue models, have emerged as promising human‐relevant platforms for recapitulating selected aspects of cardiac development, tissue organization, and function. This review evaluates current strategies for the construction of these cardiac microphysiological systems through a systematic comparison of two major approaches: development‐driven self‐organization based on intrinsic stem‐cell programs, and engineering‐driven assembly supported by bioactive materials, 3D bioprinting, and microfluidic technologies. To address key bottlenecks limiting translational utility, we outline a multidimensional maturity assessment framework encompassing sarcomeric ultrastructural organization, the fidelity of electromechanical coupling, and metabolic reprogramming toward fatty acid β‐oxidation. Furthermore, we discuss the translational applications of cardiac microphysiological systems in elucidating early cardiogenesis, modeling complex genetic and ischemic cardiovascular diseases, and enabling high‐throughput cardiotoxicity screening. Despite persistent challenges in building perfusable multi‐scale vascular networks, reducing batch‐to‐batch variability, and modeling multi‐organ crosstalk, the integration of cardiac microphysiological systems with spatial multi‐omics, next‐generation biomaterials, and artificial intelligence‐assisted culture systems may enhance their translational relevance, provided that these approaches are supported by rigorous benchmarking and cross‐laboratory validation.

**Translational Impact Statement**

This review defines a translational framework for cardiac microphysiological systems, encompassing self‐organizing human cardiac organoids and engineered cardiac tissue models, by linking construction strategies with integrated structural and functional maturation benchmarking. By clarifying how maturation status governs physiological relevance, disease‐model fidelity, and pharmacological predictiveness, it identifies the key determinants that currently limit broader deployment. The review further outlines practical routes to improve vascular support, long‐term stability, reproducibility, and scalable manufacturing, thereby supporting the development of more reliable human‐relevant cardiac MPS platforms for cardiotoxicity testing, disease modeling, and broader translational applications in cardiovascular research.

## 1 INTRODUCTION

Cardiovascular diseases (CVDs) remain a major global health challenge, serving as the leading cause of mortality and long‐term disability. As of 2023, global deaths due to CVDs surged to 19.2 million, with prevalent cases more than doubling to a record 626 million.^1^ This substantial burden underscores the complexity of cardiovascular pathology and the urgent need for sufficiently predictive human‐relevant models. In particular, the extremely limited intrinsic regenerative capacity of the adult mammalian myocardium remains a major biological obstacle to effective repair after cardiac injury and in heart failure.^2^ Consequently, elucidating the mechanisms of cardiogenesis, identifying strategies for myocardial regeneration, and screening potent cardioprotective agents remain the core mandates of cardiovascular research. However, conventional animal and 2D in vitro models often fail to fully capture the structural, cellular, and functional complexity of the human heart.^3^ This has driven an urgent demand for advanced in vitro platforms that can recapitulate key physiological and pathological features of the human heart, leading to the emergence of cardiac microphysiological systems (cardiac MPS). In this review, we focus on two major organizing paradigms within cardiac MPS: self‐organizing human cardiac organoids (COs), which arise through developmentally guided patterning, and engineered cardiac tissue models, which include multicellular cardiac microtissues, 3D‐bioprinted cardiac constructs, and microfluidic or heart‐on‐chip platforms.

Since the first demonstration of scaffold‐free, self‐organizing COs through temporally controlled developmental signaling, the field has evolved rapidly.^4^ The integration of cross‐disciplinary technologies, such as 3D bioprinting, organ‐on‐a‐chip platforms, and single‐cell omics, has further expanded the structural complexity, functional fidelity, and high‐throughput analytical capability.^5^, ^6^, ^7^ Such systems can exhibit spatial compartmentalization, multicellular integration, and electromechanically synchronized contraction, thereby recapitulating selected structural and functional features of early human cardiac tissue. These properties are particularly valuable in contexts where multicellular organization and spatial morphogenesis are central. A key challenge in the field now lies in integrating diverse construction paradigms with rigorous, multidimensional maturation assessment in order to maximize the utility of cardiac MPS in disease modeling and therapeutic evaluation. Accordingly, this review focuses on three central questions in the field: how self‐organizing COs and engineered cardiac tissue models are constructed, how their structural and functional maturation should be rigorously assessed, and how they can be translated into applications for cardiovascular disease modeling, drug evaluation, and regenerative research.

## 2 CARDIAC MPS: A NEW PARADIGM IN CARDIOVASCULAR RESEARCH

### 2.1 Limitations of current cardiac model systems

In efforts to elucidate the pathogenesis of CVDs and develop new therapeutic strategies, conventional experimental models remain indispensable but are increasingly recognized as having important limitations. Among these, animal models—particularly mouse and rat models—have provided valuable insights, yet they do not fully recapitulate key aspects of human cardiac development, physiology, and disease progression. These limitations arise in part from marked interspecies differences in cardiac physiology and molecular composition.^8^ For example, murine hearts exhibit much higher basal metabolic demand and heart rates than human hearts, and predominantly express α‐myosin heavy chain, whereas the adult human ventricle is enriched in β‐myosin heavy chain.^9^ Such differences may contribute, at least in part, to the translational gap between preclinical efficacy and clinical outcomes, including unexpected cardiotoxicity or insufficient therapeutic benefit in human trials. Likewise, although 2D culture systems are favored for their cost‐effectiveness and experimental throughput, they do not fully recapitulate the 3D microenvironment, multicellular interactions, and maturation‐promoting cues of native cardiac tissue.^10^ Collectively, the limitations of animal models and 2D culture systems restrict the ability of conventional platforms to faithfully model human cardiac development and disease. In response, cardiac MPS have gained increasing prominence as human‐relevant in vitro platforms for cardiovascular research.

### 2.2 Cardinal features of self‐organizing COs

- Developmentally guided self‐organization: Many COs possess the capacity to spontaneously orchestrate complex 3D spatial structures, a process driven by the directed differentiation and self‐organizing potential of hPSCs under defined inductive conditions. This process generates chamber‐like 3D configurations that mimic native spatial topography, facilitating coordinated cell–cell communication required for organogenesis.^11^
- Multicellular integration and heterogeneity: The native heart depends on the coordinated specification and interaction of multiple cell lineages during development and tissue organization.^12^ By recapitulating developmental trajectories, COs establish a heterogeneous cytoarchitecture that facilitates paracrine networking and ECM remodeling, thereby approximating key aspects of native tissue heterogeneity.^4^, ^12^
- Tissue‐level electromechanical function: COs exhibit core functional hallmarks of the native myocardium, including spontaneous rhythmic contractility, electrophysiological activity—characterized by action potentials and calcium transients—and predictable responsiveness to pharmacological stimuli.^11^, ^13^ These functional properties support the use of COs for studying cardiac physiology and pathophysiology.
- Recapitulation of developmental milestones: COs provide access to key stages of human cardiogenesis by recapitulating developmentally relevant events such as cavity formation, chamber specification, and multicellular cardiac patterning. Furthermore, their stage‐specific molecular marker expression provides a framework for investigating mechanisms of heart development and congenital heart disease.^11^, ^13^

Although these features are most characteristic of self‐organizing COs, engineered cardiac tissue models provide a complementary route by imposing external control over tissue geometry, extracellular matrix composition, mechanical loading, perfusion, and functional readouts. Together, these two platform classes constitute the cardiac MPS framework discussed in this review.

### 2.3 Historical evolution toward contemporary cardiac MPS platforms

Prior to the formal emergence of the “organoid” concept, cardiovascular research had already initiated its transition toward 3D paradigms. As the primordial functional organ to develop during embryogenesis, the 3D cultivation of the heart dates back to 1993, when Maltsev et al. leveraged pluripotent embryonic stem cells to differentiate into embryoid bodies harboring spontaneously beating cardiomyocytes.^14^ In the early 2000s, particularly in 2004, Zimmermann and colleagues advanced the field through engineered heart tissue, introducing scaffold‐based and mechanically constrained myocardial constructs as a complementary engineering route.^15^ By 2010, human pluripotent stem cell technology enabled the generation of functional human cardiomyocytes, substantially improving the human relevance of in vitro cardiac models.^16^ Around 2015, increasing attention was directed toward multicellular organization and self‐patterning, reflecting an emerging shift from simple cardiac aggregates to more developmentally informed 3D systems.^17^, ^18^

The year 2021 represented a major milestone in the field of self‐organizing CO research. By temporally modulating developmental signaling, Hofbauer and colleagues established scaffold‐free, self‐organizing cardioids from hPSCs, generating cavity‐containing heart‐like structures that recapitulated key features of early human cardiogenesis.^4^ By validating the feasibility of utilizing biochemical cues to orchestrate autonomous organoid assembly, this study established the foundational paradigm for COs. In subsequent years, the focus of the field transitioned toward functional refinement and preliminary applications. For instance, diverse strategies—including the implementation of mechanical strain, electrical pacing, and the modulation of the metabolic environment—have been strategically leveraged to enhance the structural and functional maturation of organoid‐derived cardiomyocytes.^3^ Meanwhile, the leveraging of patient‐derived induced pluripotent stem cells (iPSCs) and advanced gene‐editing technologies has enabled the generation of disease‐specific organoids. This progress has facilitated the in vitro modeling and mechanistic interrogation of various cardiac pathologies, most notably hereditary cardiomyopathies, within a human‐relevant genetic framework. Most strikingly, the successful engineering of “epicardioids” introduced organoid systems incorporating a functional epicardial‐like layer. This breakthrough provides a pivotal platform to study coronary angiogenesis, epicardial‐myocardial crosstalk, and the fibrotic response.^19^

The integration of interdisciplinary technologies has further expanded the engineering scope and application frontiers of cardiac MPS research. 3D bioprinting facilitates the precise spatial orchestration of diverse cell types, enabling the fabrication of engineered myocardial tissues with defined geometries and microvascular network support.^5^ Furthermore, organ‐on‐a‐chip technology integrates cardiac MPS with microfluidic systems to provide controlled fluid shear stress, mechanical strain, and nutrient perfusion. These platforms can be interconnected with other organ‐specific chips—such as liver or kidney models—to construct integrated “human‐on‐a‐chip” systems, allowing for the evaluation of systemic drug responses and inter‐organ crosstalk.^6^

In conclusion, cardiac MPS has undergone a transformative evolution, transitioning from rudimentary cellular aggregates into increasingly sophisticated functional entities through successive waves of biotechnological innovation. This paradigm shift has positioned cardiac MPS as a versatile and powerful platform with immense potential to revolutionize our understanding of developmental biology, the elucidation of complex disease mechanisms, high‐throughput drug screening, cardiotoxicity assessment, and the implementation of personalized medicine. Nevertheless, important challenges remain, including incomplete maturation, limited vascularization and perfusion, inter‐line variability, and the lack of standardized benchmarking frameworks.

## 3 TECHNICAL FRAMEWORK FOR CONSTRUCTING CARDIAC MPS

Current construction paradigms are generally classified into two broad categories: developmentally driven self‐organization, which prioritizes the intrinsic morphogenetic potential of stem cells to recapitulate developmental processes, and engineering‐driven assembly, which employs exogenous scaffolds and biophysical constraints to guide tissue architecture.

### 3.1 Self‐organizing strategies

Self‐organizing COs are generated through controlled 3D aggregation followed by sequential developmental induction, rather than through simple spontaneous spheroid culture. In most protocols, the first stage establishes mesoderm and early cardiac competence through canonical wingless‐related integration site (WNT) activation, often together with cardiogenic cofactors such as bone morphogenetic protein 4 (BMP4), activin A, or fibroblast growth factor 2 (FGF2), thereby creating the developmental context required for subsequent cardiac morphogenesis.^4^, ^20^ This early induction is then followed by timed WNT inhibition, which promotes cardiac lineage commitment and supports the emergence of myocardial tissue within the organoid. However, self‐organizing CO construction is not limited to cardiomyocyte specification. In more elaborate systems, additional signaling inputs are introduced after cardiac induction to broaden lineage output and tissue architecture. For example, matrix‐supported protocols use a preculture phase, Matrigel embedding, and sequential CHIR99021/IWP2 exposure to promote the coordinated formation of cardiac, foregut, and vascular compartments within the same 3D construct.^21^ Other approaches further incorporate vascular‐promoting cues such as vascular endothelial growth factor (VEGF) and related endothelial‐inductive signals to support the development of endothelial networks and vascular‐like organization.^22^, ^23^ In blood‐generating systems, this framework is extended by adding haemato‐endothelial and inflammatory‐supportive cues, including factors such as IL‐6, after the onset of cardiac patterning, thereby enabling the co‐development of ventricular‐like tissue, haemogenic endothelium, and hematopoietic progenitors.^23^ An overview of the principal construction paradigms of COs, together with representative differentiation and patterning timelines, is provided in Figure 1.

**FIGURE 1:**
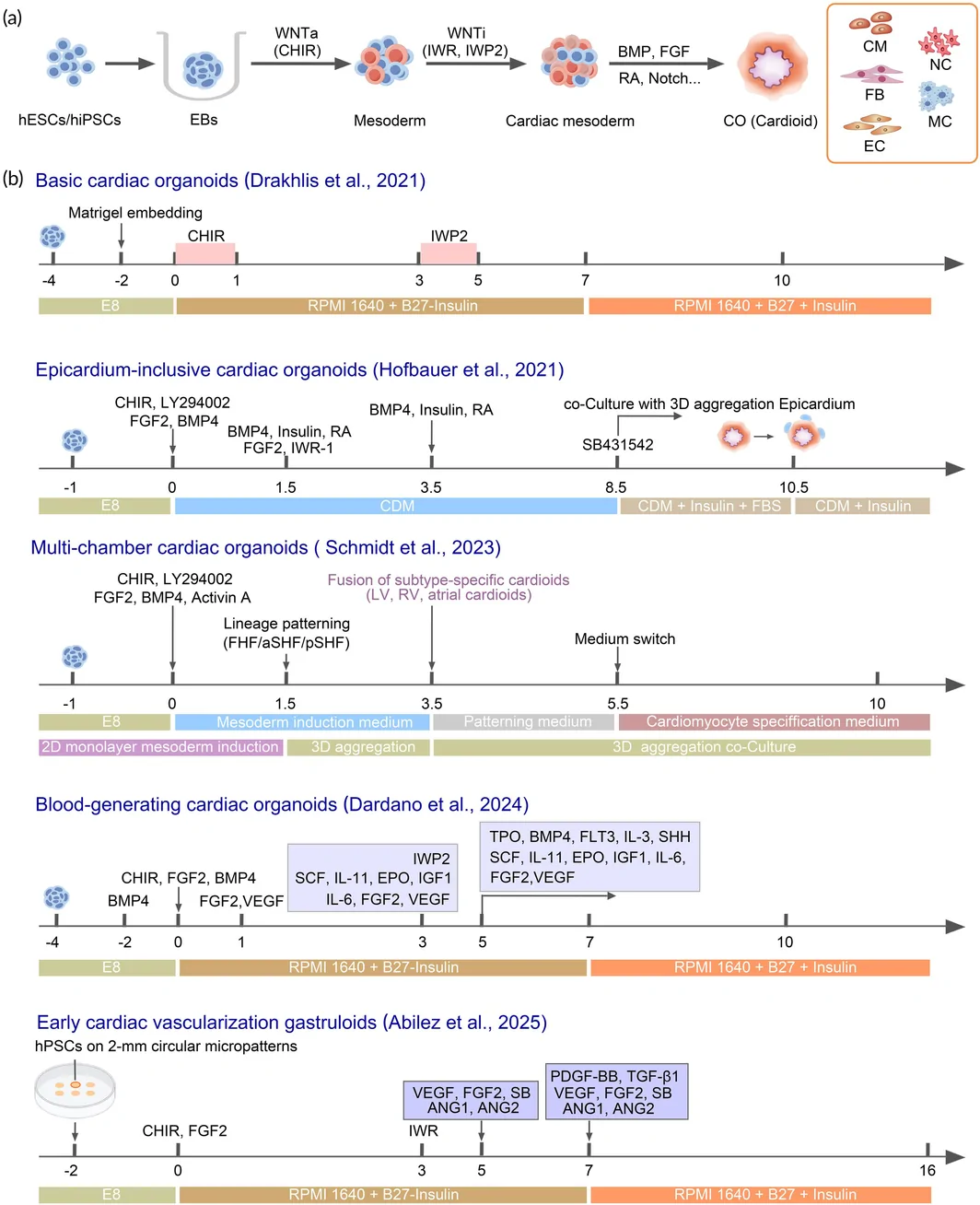
Schematic overview of self‐organizing COs generation strategies and representative induction timelines. (a) General schematic of the developmental trajectory from human embryonic/induced pluripotent stem cells (hESCs/hiPSCs) to multicellular cardioids. The protocol mimics embryonic cardiogenesis, initiating with embryoid body (EB) formation, followed by mesoderm induction via Wnt signaling activation (e.g., CHIR), and subsequent cardiac mesoderm specification through Wnt inhibition (e.g., IWR, IWP2). Maturation yields complex cardioids comprising diverse cellular populations, including cardiomyocytes (CMs), fibroblasts (FBs), endothelial cells (ECs), neural crest cells (NCs), and mural cells/macrophages (MCs). (b) Comparison of representative induction timelines for distinct self‐organizing cardiac organoid systems, including basic cardiac organoids, epicardium‐inclusive cardiac organoids, multi‐chamber cardiac organoids, blood‐generating cardiac organoids, and early cardiac vascularization gastruloids. Although all protocols rely on staged modulation of signaling pathways, they differ in matrix support, lineage‐patterning strategy, and the incorporation of vascular or hematopoietic induction cues.

Thus, in self‐organizing COs, endogenous developmental programs drive tissue formation, whereas experimentally defined culture conditions critically shape the efficiency, reproducibility, and structural complexity of the final construct. A central advantage of self‐organizing strategies lies in their high developmental fidelity, as they exploit endogenous morphogenetic programs to recapitulate early cardiogenesis. However, their broader application remains constrained by limited experimental controllability, structural heterogeneity, and substantial inter‐line, inter‐donor, and batch‐to‐batch variability. This variability may arise from differences in genetic background, epigenetic memory, pluripotency state, passage number, and responsiveness to cardiogenic signaling, as well as stochastic variation in aggregate formation and early lineage allocation. For matrix‐supported COs and engineered cardiac tissue models, reproducibility may be further influenced by lot‐to‐lot variation in Matrigel and other extracellular matrix or hydrogel formulations, which can affect differentiation efficiency, cellular composition, spatial patterning, maturation, and functional readouts.

### 3.2 Engineering‐driven assembly of engineered cardiac tissue models

Engineering‐driven construction methodologies use external interventions, including functional biomaterials and advanced bioengineering technologies, to achieve precise control over the architecture, cellular composition, and microenvironment of engineered cardiac tissue models (Figure 2). Unlike self‐organizing COs, which depend predominantly on endogenous morphogenetic programs, these platforms are characterized by greater scaffold dependence, stronger spatial controllability, and more reproducible structural organization. This distinction is supported by representative studies of self‐organizing systems^4^, ^13^, ^18^, ^20^, ^21^, ^22^, ^23^ and engineered cardiac tissue models^24^, ^25^, ^26^, ^27^, ^28^ (Table 1). Functionally, self‐organizing COs provide stronger developmental fidelity and are particularly suitable for early cardiogenesis, lineage‐patterning studies, and congenital morphogenetic defect modeling, whereas engineered cardiac tissue models provide greater controllability, reproducibility, scalability, and compatibility with quantitative functional assays and drug‐screening workflows. Importantly, engineering‐driven assembly is not limited to structural fabrication alone, but also includes subsequent regulation of the local niche, tissue maturation, and functional conditioning.

**FIGURE 2:**
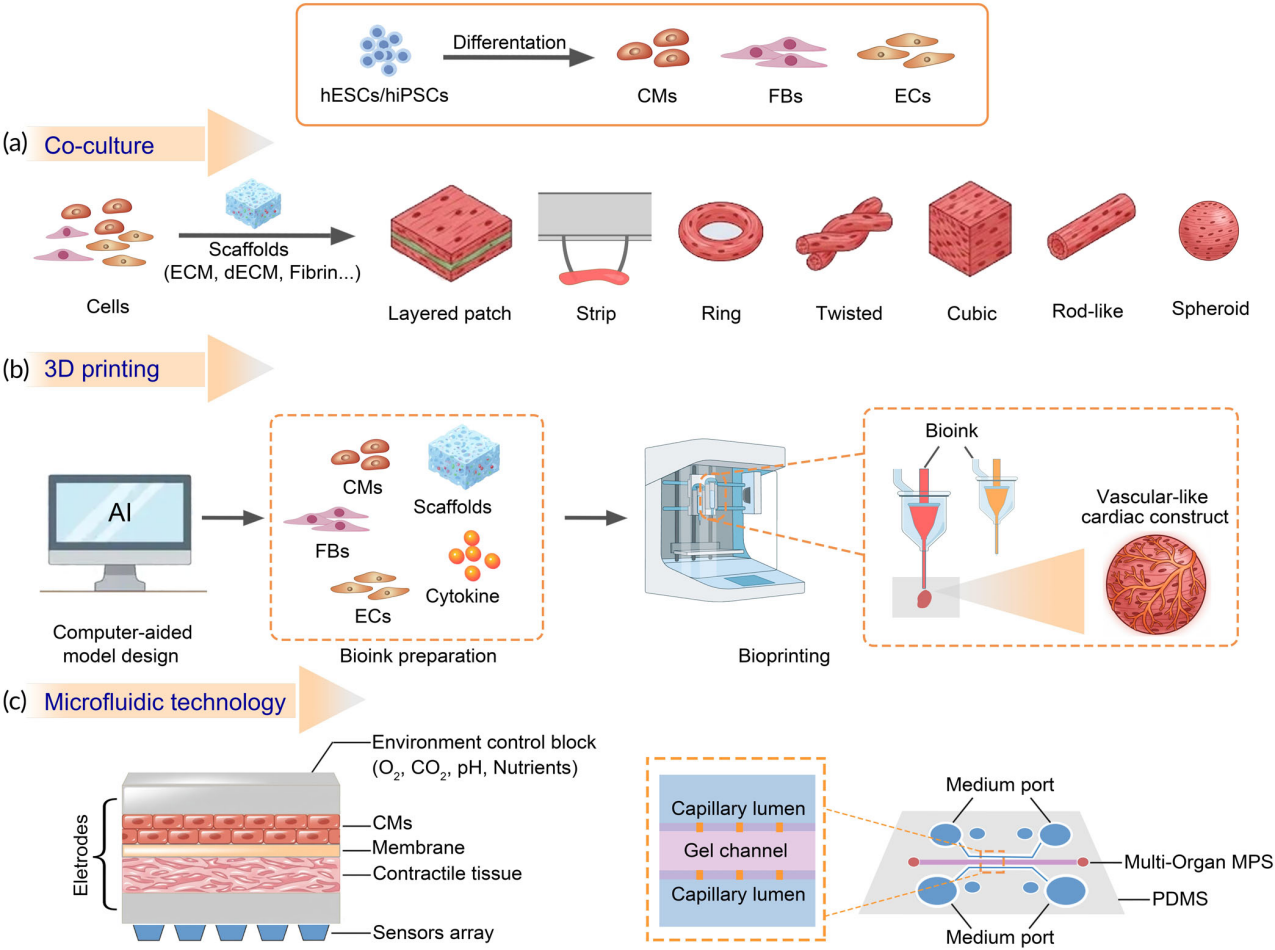
Engineering‐driven strategies for constructing and integrating engineered cardiac tissue models. Differentiated derivatives from human pluripotent stem cells, including cardiomyocytes (CMs), fibroblasts (FBs), and endothelial cells (ECs), serve as the cellular building blocks. (a) Co‐culture with biomaterial scaffolds: Cells are seeded onto or encapsulated within natural or synthetic scaffolds (e.g., ECM, dECM, fibronectin) to generate engineered heart tissues (EHTs) with diverse tailored geometries, such as layered patches, strips, rings, twisted macroscopic bundles, cubes, rod‐like structures, and spheroids. (b) 3D bioprinting: Computer‐aided model design (AI‐assisted) guides the precise spatial deposition of customized bioinks—formulated with specific ratios of CMs, FBs, ECs, supportive scaffolds, and cytokines—to fabricate vascular‐like cardiac constructs. (c) Microfluidic technology (Heart‐on‐a‐chip): Microfluidic systems enable precise control over the cellular microenvironment. Left: A sophisticated chip design integrating an environment control block (regulating O_2_, CO_2_, pH, and nutrients), layered cellular constructs (CM, membranes, and contractile tissues), and embedded electrode/sensor arrays for real‐time functional readouts. Right: A multi‐organ MPS polydimethylsiloxane (PDMS) platform featuring perfusable capillary lumens, gel channels, and medium ports to simulate fluid dynamics and facilitate dynamic tissue crosstalk.

**TABLE 1:** Key distinctions between self‐organizing COs and engineered cardiac tissue models.

Characteristic	Self‐organizing COs	Engineered cardiac tissue models
Cell source and construction principle	hPSC self‐differentiation under spatiotemporal cues	Defined assembly of selected cell types with controllable composition
Matrix/Scaffold dependence	Often scaffold‐free or minimally matrix‐supported	Frequently dependent on biomaterials for structural guidance
Experimental controllability	Limited external control over cell composition and spatial patterning	High control over cell composition, geometry, and spatial organization
Structural organization and anisotropy	Intrinsic tissue patterning can emerge, but anisotropy and alignment are often variable and incompletely controlled	Structural alignment and anisotropy can be introduced more reproducibly through scaffold design, micropatterning, or bioprinting
Functional properties	Spontaneous beating and emergent electromechanical activity	More controllable electrophysiology, contractility, and sensor‐compatible readouts
Developmental fidelity	Stronger recapitulation of early morphogenesis, lineage co‐development, and multicellular developmental interactions	Often reduced endogenous developmental spontaneity, but allows targeted reconstruction of selected tissue‐level features
Reproducibility and standardization	More susceptible to batch‐to‐batch variability and structural heterogeneity	More amenable to standardization, parameter control, and inter‐experimental reproducibility
Scalability and throughput	Relatively simple culture workflow, but variability may limit scalability for large comparative studies	Greater potential for standardized manufacturing and parallelization, although fabrication complexity may reduce throughput in some systems
Main application	Human cardiogenesis, lineage‐patterning studies, chamber‐like morphogenesis, and congenital defect modeling	Reproducible functional assays, controlled disease modeling, cardiotoxicity assessment, drug‐response testing, and preliminary evaluation of reparative tissue designs
Key advantages	High developmental fidelity, intrinsic multicellular crosstalk, and relatively simple culture setup	Precise structural design, reproducibility, spatial control, and scalability
Limitations	High structural variability, limited control of geometry and cell positioning, and slow or inconsistent vascularization	Reduced developmental spontaneity, fabrication‐related stress, bioink/material constraints, and higher technical requirements

*Note*: Representative references for self‐organizing COs include References 4, 13, 18, 20, 21, 22, 23, whereas representative references for engineered cardiac tissue models include References 25, 26, 27, 28.

#### 3.2.1 Selection and design of biomaterials

Biomaterials are fundamental to the construction of engineered cardiac tissue models, particularly engineered cardiac tissue models, as their biochemical composition and biophysical properties shape the local microenvironment that governs cell adhesion, survival, proliferation, lineage commitment, and multicellular organization.^27^ Beyond structural support, they also influence matrix remodeling and cell‐matrix signaling, thereby affecting tissue stability and developmental relevance.^24^, ^27^ Given this diversity, biomaterial selection has evolved across naturally derived, synthetic, composite, conductive, and mechanotopographic platforms—each offering distinct advantages in recapitulating the native cardiac microenvironment. Table 2 summarizes representative biomaterial systems and design strategies relevant to cardiac MPS, with emphasis on their engineering applications, advantages, and limitations. The additional process‐dependent requirements imposed by bioprinting on cell‐laden formulations are discussed separately in the following section on bioink design.

**TABLE 2:** Comparative summary of biomaterials and biophysical design strategies for Engineered cardiac tissue models.

Category	Material	Applications	Advantages	Limitations	Ref
Naturally derived biomaterials	Collagen	Biomimetic matrix for promoting vascularization	Biocompatible and biodegradable; native integrin‐binding motifs support endothelial adhesion and vascular morphogenesis	Low mechanical strength; poor thermal and solvent stability; poorly controlled degradation kinetics; source‐dependent batch variability	^29^
Naturally derived biomaterials	Fibrin	Matrix for endothelial lumen formation and vascularized engineered myocardial tissue construction	Biocompatible and biodegradable; ECM‐like nanofibrous matrix; tunable in situ gelation; favorable ductility and elasticity	Rapid degradation; low standalone mechanical strength; limited shape stability; source variability and pathogen‐transmission concerns for plasma‐derived products	^29^, ^30^
Naturally derived biomaterials	Hyaluronic acid‐based hydrogels	Pro‐angiogenic factor delivery and vascular‐microenvironment modulation	Biocompatible; readily modifiable and crosslinkable; tunable mechanics and degradation; modulates inflammatory and angiogenic cues	Molecular‐weight‐dependent bioactivity; possible anti‐angiogenic effects of high‐MW HA; low stability and rapid enzymatic degradation of native HA	^29^
Naturally derived biomaterials	Gelatin‐based hydrogels	Drug delivery, tissue engineering, wound dressing, and 3D cell culture	Biocompatible and biodegradable; supports cell adhesion and proliferation; highly absorbent; readily modifiable with tunable mechanical properties	Poor native mechanical and physiological stability; requires crosslinking; performance is processing‐dependent	^31^, ^32^
Naturally derived biomaterials	Decellularized extracellular matrix (dECM) hydrogels	Scaffolds and bioinks for engineered heart tissues, cardiac microphysiological models, and cardiotoxicity testing	Preserve tissue‐specific ECM proteins and biochemical cues; porous and biocompatible; support cardiomyocyte organization and functional maturation; exhibit shear‐thinning behavior suitable for bioprinting	Native dECM has limited mechanical strength and electrical conductivity, with a modulus mismatch relative to myocardium; composition may be partially altered during decellularization; batch‐to‐batch variability	^33^
Synthetic biomaterials	PEG hydrogels	Tunable matrices for cell‐adhesion and mechanobiology studies	Independently tunable stiffness and RGDS content; biocompatible and chemically defined	Lacks intrinsic cell‐adhesion sites; requires functionalization; cell responses depend on matrix stiffness and cell type	^34^
Synthetic biomaterials	PCL	Aligned or anisotropic scaffold for cardiomyocyte organization and tissue architectural guidance	Good mechanical strength; suitable for anisotropic structural design	Hydrophobic and lacks intrinsic cell‐recognition sites; limited cardiomyocyte attachment and spreading; relatively high stiffness may restrict cardiac contraction	^35^
Synthetic biomaterials	PLGA	Biodegradable carrier for controlled delivery of angiogenic factors in cardiac engineering	Controlled release capability; biodegradable	Primarily a delivery vehicle rather than a biomimetic matrix	^36^
Composite and hybrid biomaterials	Alginate/gelatin composite hydrogels	3D‐printed, myocardium‐inspired scaffolds for cardiac tissue engineering	Combine gelatin bioactivity with alginate printability and structural support	Requires optimization of printability, mechanics, and biological performance	^37^
Advanced biomimetic hydrogels	Self‐assembling polypeptide/ peptide hydrogels	Tunable 3D matrices for cell culture and tissue engineering	Defined composition; tunable bioactivity and stiffness; high design flexibility	Assembly‐ and sequence‐dependent properties	^38^, ^39^
Conductive biomaterials	Electroconductive hydrogels incorporating carbon‐based nanomaterials or conductive polymers	Electrically supportive matrices for cardiac tissue engineering	Enhance electrical communication and functional integration	Potential cytotoxicity, formulation complexity, and dose‐dependent effects	^40^, ^41^
Biophysical design strategies	Myocardium‐ matched stiffness scaffolds	Modeling physiological or pathological cardiac microenvironments, including fibrosis and scar‐like stiffening	Support more physiologically relevant mechanoregulation and contractile behavior	Responses are stiffness‐, cell‐, and tissue‐dependent; nonphysiological stiffness may impair function; stiffness alone is insufficient to model fibrosis	^42^, ^43^, ^44^
Topographical design strategies	Aligned electrospun fibrous scaffolds	Guiding cardiomyocyte alignment and anisotropic tissue organization	Promote oriented cell organization and structural anisotropy	Alignment alone may be insufficient to induce full maturation	^35^, ^46^
Topographical design strategies	Micropatterned scaffolds and anisotropic cardiac sheets	Cardiomyocyte alignment and anisotropic contraction/conduction	Better recapitulate anisotropic myocardial architecture and function	Fabrication‐ and pattern‐dependent; incomplete maturation	^45^, ^47^, ^48^, ^49^

##### Naturally derived biomaterials

Natural polymers are prioritized for their innate bioactivity and biomimetic cues. Collagen and fibrin offer exceptional biocompatibility and tunable mechanical stiffness via cross‐linking density, providing the necessary mechanical resistance for synchronized cardiomyocyte contraction.^29^, ^30^ Hyaluronic acid and gelatin are frequently modified to create stimuli‐responsive or bioactive hydrogels (e.g., via RGD motifs), supporting non‐invasive cell recovery and targeted adhesion.^31^, ^32^ While Matrigel remains the gold standard due to its growth factor‐rich milieu, which is highly effective in supporting the initial self‐assembly and phenotypic maintenance of cardiac cells, its tumor‐derived nature and lot‐to‐lot variability hinder clinical translation.^21^ Consequently, decellularized ECM has emerged as a compelling alternative, preserving tissue‐specific biochemical profiles to significantly promote cardiac lineage‐relevant microenvironmental signaling and tissue‐specific support.^33^

##### Synthetic biomaterials

Synthetic polymers offer superior controllability and mechanical robustness compared with natural materials, making them attractive for engineered cardiac constructs. PEG is widely used as a bio‐inert and mechanically tunable platform, and PEG‐based hydrogels have been engineered with independently adjustable stiffness and other matrix parameters for mechanobiology studies.^34^ PCL, particularly in electrospun or anisotropic scaffold formats, can provide topographic guidance for cardiac cell alignment and tissue organization.^35^ In addition, PLGA is frequently used as a biodegradable carrier for the controlled release of pro‐angiogenic factors such as VEGF.^36^ A current trend in cardiac engineering is the development of hybrid systems that graft bioactive natural motifs onto synthetic backbones, thereby seeking a synergistic balance between structural durability and biological functionality.

##### Composite and hybrid biomaterials

Composite hydrogels, such as those combining gelatin and sodium alginate, leverage the inherent cell‐adhesion properties of gelatin together with the favorable biocompatibility and matrix‐forming capacity of sodium alginate. These hybrid materials can provide a permissive matrix for multicellular cardiac assembly and spatially organized engineered tissue formation.^37^ For more precise control over the cellular microenvironment at the molecular level, self‐assembling peptide hydrogels employ specific amino acid sequences to govern bioactivity and mechanical stiffness.^38^ Notably, low‐molecular‐weight dipeptide hydrogels have garnered significant attention for their structural stability and synthetic accessibility in drug delivery and ECM mimicry.^38^ Advanced self‐assembling peptide platforms, such as PeptiGel®, allow fine‐tuning of composition and mechanical stiffness to support 3D cardiac cell and tissue culture and may therefore provide a more controllable synthetic matrix for engineered cardiac tissue models.^39^

##### Integration of conductive biomaterials

Myocardial tissue is characterized by intrinsic electroactivity, with its coordinated functionality relying on seamless intercellular electrical coupling. Likewise, the functional performance of engineered cardiac tissue models depends on efficient electrical communication among cardiomyocytes. However, the inherent insulating properties of conventional natural and synthetic hydrogels can limit excitation propagation and coordinated contraction in three‐dimensional cardiac constructs. To address this challenge, the incorporation of conductive nanomaterials—such as carbon nanotubes, graphene, or conductive polymers like PEDOT:PSS—into the hydrogel matrix has emerged as a promising bioengineering strategy for improving electrical signal propagation and contraction synchrony in engineered cardiac tissues.^40^ By providing low‐resistance pathways, these materials can facilitate excitation propagation and, in some settings, enhance the expression and distribution of gap junction proteins, particularly Cx43.^40^, ^41^ Such effects may support more synchronized electromechanical activity, although conductive modification alone does not ensure comprehensive cardiac tissue maturation. Notably, the concentration of these materials must be carefully optimized to balance enhanced conductivity against potential cytotoxicity and adverse alterations in matrix properties. Such conductive scaffolds are particularly advantageous for disease modeling, especially when engineered cardiac tissue models are intended to recapitulate conduction abnormalities associated with myocardial injury.^40^, ^41^

##### Modulation of scaffold mechanical stiffness and topographic features

The heart is an organ subjected to continuous dynamic mechanical loading; as such, the biophysical properties of the scaffolding material are paramount for the microenvironmental regulation and functional organization of engineered cardiac tissue models. The Young's modulus of the biomaterial should be precisely tuned to match the relevant myocardial microenvironment. Native‐like myocardium has been reported to fall approximately within the range of 10–30 kPa, whereas infarcted or fibrotic myocardium is substantially stiffer, commonly reaching 35–70 kPa or higher.^42^ Accordingly, when constructing engineered cardiac tissue models of myocardial infarction or fibrosis, scaffold stiffness can be adjusted to reflect the significant tissue hardening caused by excessive collagen deposition and cross‐linking.^42^ Substrates that are excessively compliant (too soft) fail to support effective contractile force transmission, whereas supra‐physiological rigidity (too stiff) can mechanically impede rhythmic contractility and induce cellular stress.^42^, ^43^ Importantly, the effects of matrix stiffness are not limited to passive mechanical support, but may also extend to cell‐state regulation through mechanically regulated transcriptional and epigenetic remodeling. In cardiac valve interstitial cells, pathological extracellular matrix stiffening has been shown to promote myofibroblast activation in association with chromatin remodeling and increased pro‐fibrotic gene expression, suggesting that abnormal mechanical cues can actively drive disease‐relevant cellular phenotypes rather than merely accompany them.^44^ This concept is relevant to the design of engineered cardiac tissue models intended to reproduce fibrotic remodeling and other mechanically driven pathological states.

Beyond mechanical matching, the integration of topographical cues is essential for structural organization in engineered cardiac tissue models. Fibrous scaffolds fabricated via electrospinning or micropatterning technologies provide structural anisotropy, offering critical contact guidance to the seeded or encapsulated cells.^45^, ^46^, ^47^ These cues induce the longitudinal alignment of cardiomyocytes and enhance sarcomeric organization, thereby helping to recapitulate the anisotropic architecture and directed conduction characteristic of native cardiac tissue.^45^, ^47^, ^48^ This aligned configuration may facilitate directional impulse propagation and improve the electrophysiological relevance of engineered cardiac tissue models for drug screening applications, with potential relevance for the design of functionally anisotropic self‐organizing COs.^48^, ^49^

Overall, biomaterial selection for engineered cardiac tissue models should be guided by a balance among biological relevance, engineering controllability, scalability, and translational feasibility. Naturally derived biomaterials provide intrinsic bioactivity, biocompatibility, cell‐adhesive cues, and tissue‐relevant extracellular matrix signals, thereby offering high biological relevance; however, their broader application may be limited by batch‐to‐batch variability, less‐defined composition, source dependence, restricted mechanical tunability, and regulatory concerns. In contrast, synthetic biomaterials offer more defined chemistry, tunable mechanical and degradation properties, improved reproducibility, and greater compatibility with scalable manufacturing, but they often lack intrinsic biological instructiveness and therefore require functionalization with adhesive ligands, extracellular matrix motifs, or growth‐factor‐binding components. Composite or hybrid biomaterials seek to combine the biological instructiveness of natural matrices with the controllability and manufacturability of synthetic systems, although their increased compositional complexity may complicate formulation reproducibility, quality control, and translational characterization. Accordingly, no single biomaterial class is universally optimal, and material selection should be tailored to the intended application, manufacturing strategy, and translational requirements. In addition, conductive components can enhance electrical signal propagation and contraction synchrony, whereas matrix stiffness and topographical cues should be tailored to the intended physiological or pathological context to support appropriate contractile behavior, cardiomyocyte alignment, and anisotropic tissue organization.

#### 3.2.2 Bioprinting‐based engineered assembly

As an emerging paradigm in additive manufacturing, bioprinting enables the precise spatial deposition of bioinks—comprising cells, growth factors, and biomaterials—in a layer‐by‐layer manner, thereby offering high spatial control and versatility for the fabrication of engineered cardiac tissue models.^50^ Bioprinting modalities are primarily categorized into three major classes based on their underlying deposition mechanisms: extrusion‐based bioprinting, photopolymerization‐based bioprinting (also referred to as vat photopolymerization bioprinting, VPB, encompassing stereolithography and digital light processing), and inkjet‐based bioprinting.^51^

##### Design principles of bioinks

Unlike the broader selection of biomaterial scaffolds, bioink design for 3D‐bioprinted cardiac constructs should prioritize printability, cross‐linking control, cytocompatibility, and cardiac‐relevant bioactivity.^52^, ^53^ As a cell‐laden formulation, a bioink must maintain sufficient printing fidelity during deposition while preserving structural stability and a supportive microenvironment after printing.^52^, ^53^, ^54^ It is important to distinguish biomaterial scaffolds from bioinks. In conventional tissue engineering, scaffolds such as electrospun PCL meshes or PLGA‐based carriers can be fabricated under cell‐free conditions and subsequently seeded with cells; therefore, their processing may involve organic solvents, elevated temperatures, electrospinning, or other conditions that would be incompatible with direct cell encapsulation. By contrast, bioinks are printable formulations containing living cells and must preserve cell viability during extrusion, photopolymerization, or other printing processes. Cell‐free printable formulations should be distinguished as biomaterial inks. Thus, materials used as structural scaffolds or drug‐delivery carriers should not be assumed to be directly interchangeable with bioinks for cell‐laden cardiac bioprinting.

For extrusion‐based bioprinting, desirable bioinks generally exhibit shear‐thinning behavior, appropriate yield stress, and rapid structural recovery, which help reduce shear‐associated cell damage and improve shape retention.^52^, ^53^, ^54^ For light‐based bioprinting, cross‐linking kinetics, photoinitiator selection, and light exposure conditions must be carefully controlled to achieve geometric accuracy without substantially reducing cell viability.^55^, ^56^ Beyond printability, cardiac bioinks should provide biochemical and biophysical cues that support cell adhesion, spatial organization, and heterocellular interactions, while maintaining mechanical compliance compatible with soft myocardial‐like tissues.^53^, ^54^, ^56^, ^57^ In this context, cardiac decellularized extracellular matrix‐derived bioinks and hybrid formulations combining bioactive natural polymers with mechanically supportive synthetic components have been widely explored to balance biomimetic signaling, printability, and structural stability.^53^, ^54^, ^56^, ^57^ Overall, bioink design for cardiac bioprinting should be considered a multidimensional optimization process in which printability, structural support, and relevance to the cardiac microenvironment are balanced according to the printing modality and target construct.^52^, ^53^, ^54^, ^55^, ^56^, ^57^

##### Distinct advantages and persistent bottlenecks of bioprinting

1. Spatial Patterning of Endothelialized Vascular‐like Structures: Bioprinting enables high‐fidelity control over the spatial distribution of diverse cell populations and biomaterials, facilitating the construction of engineered cardiac constructs with predefined cellular and tissue architectures. For instance, bioprinted microfibrous scaffolds incorporating endothelial components have been used to engineer endothelialized myocardium and heart‐on‐a‐chip platforms with coordinated contractile behavior.^58^
2. Improved Geometric Control for Cardiac Tissue Modeling: This technology enables the fabrication of structurally defined cardiac constructs with greater geometric reproducibility than conventional self‐organizing approaches. This feature is advantageous for disease modeling, functional interrogation, and drug screening, although current evidence remains stronger for engineered cardiac tissues and organoid‐based screening platforms than for fully chamber‐specific bioprinted cardiac models.^59^, ^60^
3. Integration of Multicellular Systems: Bioprinting also facilitates the incorporation of multiple cardiac‐relevant cell populations within a defined 3D construct. For instance, constructs containing cardiomyocytes, fibroblasts, and endothelial cells have been developed to preserve heterocellular interactions, thereby better reflecting the cellular heterogeneity and intercellular crosstalk of native myocardium.^61^
4. Controllable Structural Anisotropy: Bioprinting allows for the fabrication of scaffolds with anisotropic topographic cues, which can guide the directional alignment of cardiomyocytes and improve biomimetic tissue organization.^62^ However, these cues mainly contribute to structural organization, whereas electromechanical stimulation and biochemical medium modulation remain necessary for broader maturation.

Despite these promising capabilities, the field still faces important challenges. A persistent trade‐off between printing resolution and cell viability remains a primary concern, because higher‐resolution strategies often require smaller nozzles or greater energy input, which may increase mechanical or photothermal stress on encapsulated cells.^63^ Furthermore, developing ideal bioinks remains challenging, as materials must simultaneously satisfy the conflicting demands of shear‐thinning behavior, shape fidelity, cytocompatibility, and tissue‐relevant mechanical properties.^64^, ^65^ Current printing strategies can generate endothelialized channels or perfusable microvascular‐like features in selected constructs. However, constructing functional, hierarchical vascular networks—spanning from larger vessels to capillary‐scale structures—remains technically difficult.^64^

#### 3.2.3 Microfluidic technology

Microfluidic technology has emerged as an important engineering strategy for advancing cardiac MPS by enabling precise control of the in vitro microenvironment at microscale resolution.^66^, ^67^, ^68^, ^69^ Compared with conventional static culture systems, microfluidic platforms support dynamic regulation of fluid flow, mass transport, biomechanical stimulation, and inter‐compartment communication, thereby providing a more physiologically relevant setting for cardiac tissue organization, maturation, and functional assessment.^66^, ^67^, ^68^, ^69^ In this context, their roles can be considered from two complementary aspects: as fabrication platforms that employ channel‐ or chamber‐based architectures to support tissue assembly, perfusion, and integrated sensing, and as functional platforms that enable dynamic microenvironmental regulation, inter‐organ crosstalk, real‐time monitoring, and biomimetic vascularization. Together, these features enhance the structural fidelity, functional complexity, and translational relevance of cardiac MPS.^69^

##### Fabrication methodologies

- Channel‐based perfusion systems (microenvironment regulation)

The core of these chips is the reproduction of controlled fluid‐flow pathways through microscale channel networks. They are typically designed using oxygen‐permeable polymeric materials to construct interconnected micro‐pathways. Cells are encapsulated in hydrogels (e.g., collagen or fibrin) and loaded into the channels to form perfusable 3D cardiac tissue compartments. Continuous perfusion flow provides nutrients and oxygen to the tissue while exposing the constructs to flow‐mediated mechanical stimulation.^70^, ^71^ Exemplifying this approach, a perfused microphysiological system was engineered for the 3D co‐culture of human induced pluripotent stem cell‐derived cardiomyocytes (hiPSC‐CMs) within a fibrin matrix. Such dynamic perfusion platforms have been shown to promote vascular‐like endothelial organization and to support more physiologically relevant myocardial function.^72^ Beyond simple channel and chamber architectures, microfluidic systems can also be combined with hydrogel‐based culture matrices to better coordinate dynamic perfusion with local biochemical and biophysical support, thereby improving the physiological relevance of engineered cardiac tissues.

ii Chamber‐based integrated systems (functional monitoring and electrophysiological readout)

These systems focus on constructing cardiac tissue within independent micro‐culture chambers. Their primary advantage lies in the ability to integrate sensing elements in situ, enabling real‐time monitoring of cardiac tissue‐level functions. Microelectrode arrays or flexible cantilever structures are often integrated within the chambers. By anchoring the engineered tissues or organoid‐like constructs in specific chambers, electrophysiological activity and contractile behavior can be monitored in real time.^73^ In addition, microfluidic chamber‐based platforms can be extended to more complex co‐culture settings, including multi‐organ MPS, thereby enabling the maintenance of organ‐specific function and the study of inter‐organ communication under controlled flow conditions.^74^

##### Microfluidic‐enabled functional platforms

- Controllable dynamic microenvironments

Microfluidic platforms provide continuous perfusion, which recapitulates the hemodynamic mechanical stimuli essential for cardiomyocyte maturation and vascularization. For instance, a heart‐kidney co‐culture MPS has demonstrated the capability to sustain the viability of cardiac microtissues and renal organoids for over 72 h under dynamic perfusion. This setup preserves organ‐specific phenotypic integrity, including the rhythmic beating of cardiomyocytes and organ‐specific structural and functional features in the renal compartment.^74^ Furthermore, these platforms allow for precise modulation of critical physiological parameters, such as fluid shear stress, mechanical strain (tensile/compressive), oxygen gradients, and pH conditions.

ii Recapitulating inter‐organ crosstalk

Microfluidic chips facilitate the fluidic coupling of distinct tissue and organ‐model compartments, enabling the construction of multi‐organ‐on‐a‐chip systems to investigate systemic physiological and pathological interactions. For example, a “heart‐kidney unit” model—constructed by connecting cardiac and renal chambers via channels—can be used to study inter‐organ communication and systemic drug responses, providing a platform for systemic pharmacology research.^74^

iii High‐throughput profiling and real‐time sensing

Microfluidic platforms provide a promising basis for miniaturized and potentially scalable screening systems. Concurrently, the integration of on‐chip biosensors enables the non‐invasive, real‐time monitoring of functional parameters, including electrophysiological activity and contractile behavior in cardiac MPS.^75^

iv Biomimetic vascularization

Within microfluidic channels, vascular endothelial cells exhibit flow‐induced alignment parallel to the direction of the medium flow. This phenomenon effectively mimics the native vascular architecture under hemodynamic flow, and endothelial layers cultured on aligned fibrin matrices have been shown to exhibit enhanced barrier integrity.^76^, ^77^

Collectively, the integration of biomaterial‐based scaffolding, precision biofabrication, and dynamic microfluidic conditioning has substantially expanded the technological framework for generating and refining cardiac MPS. However, increasing structural sophistication alone does not necessarily confer physiological relevance.

## 4 ASSESSMENT OF STRUCTURAL AND FUNCTIONAL MATURATION IN CARDIAC MPS

The maturation status of cardiac MPS is a key determinant of their ability to recapitulate the structural and physiological features of native myocardial tissue. A rigorous assessment of maturation therefore requires a multidimensional framework integrating both structural and functional benchmarks. Structural maturation encompasses tissue architecture, spatial organization, multicellular composition, and developmental‐stage molecular validation, whereas functional maturation is reflected in electrophysiological behavior, excitation‐contraction (E‐C) coupling, and metabolic adaptation. Importantly, these dimensions are not independent: functional maturation represents the physiological manifestation of progressive structural refinement. Systematic quantification of these parameters is therefore essential for evaluating the physiological relevance of cardiac MPS and defining their translational utility (Figure 3). The principal maturation metrics, representative analytical approaches, and commonly used benchmark features are summarized in Table 3.

**FIGURE 3:**
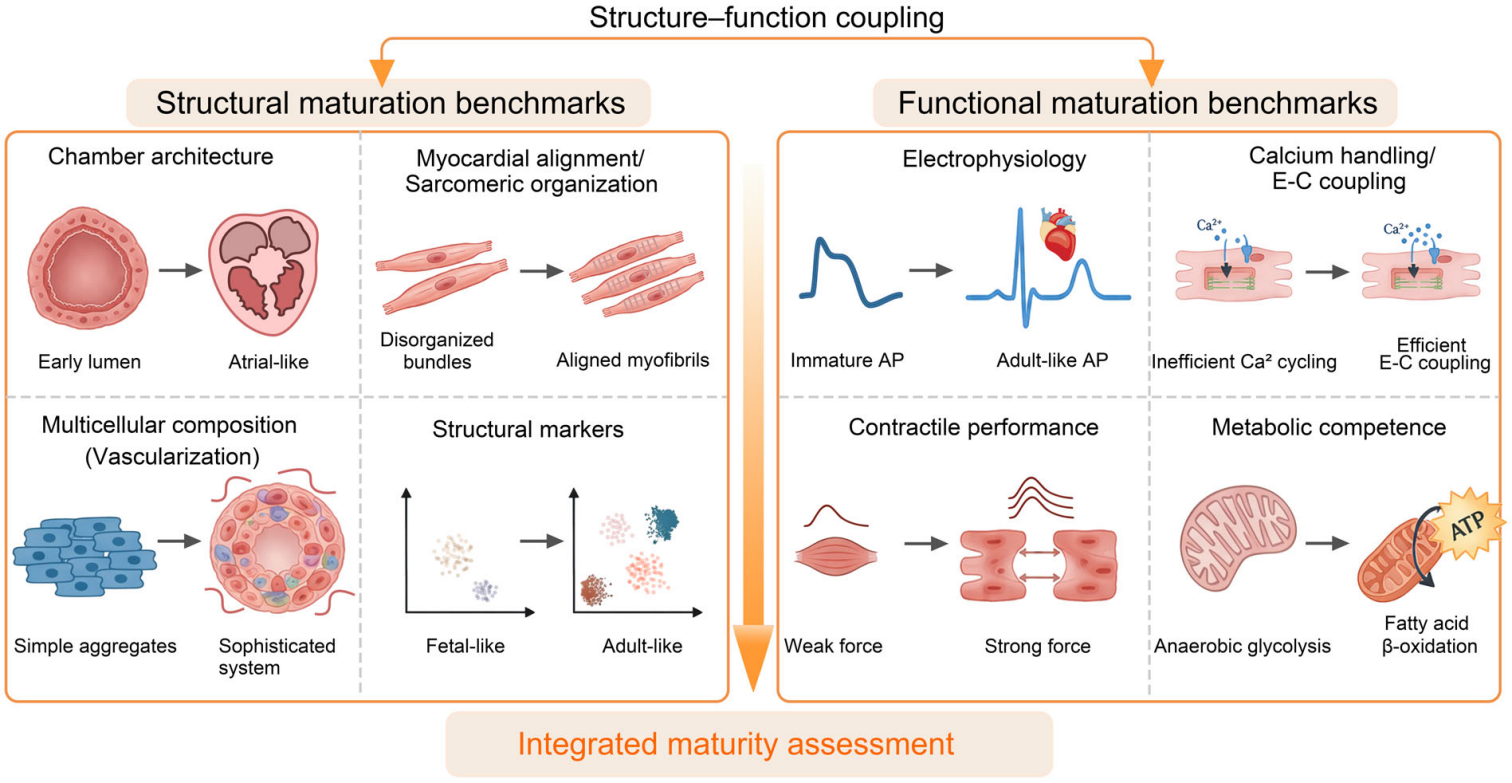
Integrated framework for evaluating the maturation of cardiac MPS. This figure illustrates an integrated structure–function coupling framework used to evaluate the maturation of cardiac MPS. Structural maturation is assessed through key features such as chamber architecture, myocardial alignment, sarcomeric organization, multicellular composition with vascularization, and the expression of stage‐specific structural markers. Functional maturation is characterized by electrophysiological properties, calcium handling and excitation‐contraction coupling, contractile performance, and the capacity for metabolic reprogramming. Together, these parameters provide a multifaceted assessment of cardiac MPS maturation, encompassing both structural integrity and functional competence.

**TABLE 3:** Key maturation metrics, representative analytical approaches, and maturity benchmarks for cardiac microphysiological systems.

Assessment domain	Biomarkers or functional readouts	Analytical approaches	Representative maturity benchmarks
Myofibrillar and ultrastructural maturation	Sarcomere length and periodicity; cardiomyocyte alignment; Z‐disc/A‐band organization; junctional Cx43 localization	Immunostaining and confocal microscopy; TEM; histology; live sarcomere imaging	Sarcomere length approaching approximately 2.2 μm; increasingly aligned and periodic myofibrils; ordered sarcomeric bands and polarized junctional Cx43
Chamber‐like morphogenesis and spatial organization	Lumen or cavity formation; internal connectivity; regional lineage patterning	Histology; XPCT; OCT; longitudinal 3D imaging	Progressive cavity formation and spatial compartmentalization; atrial‐like or ventricular‐like regional patterning where applicable; platform‐specific to self‐organizing cardiac organoids
Multicellular and extracellular‐matrix architecture	Cell‐type composition and spatial distribution; vascular organization; ECM deposition and patterning	Immunostaining and 3D imaging; scRNA‐seq; spatial profiling; ECM or proteomic analysis	Organized non‐myocyte populations; improved vascular and stromal organization; more native‐like collagen, fibronectin, and ECM distribution
Molecular maturation	TNNI1‐to‐TNNI3 transition; sarcomeric isoform remodeling; adult‐associated transcriptional and proteomic programs	qPCR; bulk RNA‐seq or scRNA‐seq; Western blot; proteomics; multiplexed immunofluorescence	TNNI3/(TNNI1 + TNNI3) ratio up to ~18%; coordinated adult‐associated isoform remodeling; greater similarity to in vivo cardiac molecular references
Electrophysiology	Resting membrane potential; action‐potential amplitude and morphology; APD50/APD90; upstroke velocity; conduction velocity; beat‐rate variability	Patch clamp; MEA; voltage imaging; optical mapping; multiparametric optical electrophysiology	More negative resting membrane potential; conduction velocity of ~25 cm/s; appropriate β‐adrenergic responsiveness
Calcium handling	Calcium‐transient amplitude; time to peak; decay time or decay constant; spatial uniformity; sarcoplasmic‐reticulum contribution; T‐tubule–dyad organization	Live Ca^2+^ imaging; simultaneous voltage–Ca^2+^ imaging; optical mapping; immunostaining or ultrastructural analysis	Larger and more spatially uniform Ca^2+^ transients; faster rise and decay kinetics; increased sarcoplasmic‐reticulum contribution; improved dyadic organization
Contractility	Contractile force or stress; contraction amplitude; contraction and relaxation kinetics; rhythmicity	Force sensors; micropost or cantilever systems; impedance measurements; video‐based motion analysis; mechanical testing	Increased force or contractile stress to 11.28 mN/mm^2^; faster and coordinated contraction–relaxation; stable rhythmic mechanical output
Metabolism	OCR; ECAR; OCR/ECAR ratio; oxidative phosphorylation; fatty‐acid β‐oxidation; mitochondrial organization and substrate utilization	Seahorse extracellular‐flux analysis; respirometry; metabolomics; mitochondrial imaging or TEM; substrate‐utilization assays	Increased OCR and OCR/ECAR ratio; reduced glycolytic dependence; increased fatty‐acid oxidation, mitochondrial density approaching ~30%, and substrate flexibility

Abbreviations: APD50 and APD90, action‐potential duration at 50% and 90% repolarization, respectively; Cx43, connexin 43; ECAR, extracellular acidification rate; ECM, extracellular matrix; MEA, multielectrode array; OCR, oxygen‐consumption rate; OCT, optical coherence tomography; scRNA‐seq, single‐cell RNA sequencing; TEM, transmission electron microscopy; XPCT, X‐ray phase‐contrast tomography.

### 4.1 Structural maturation benchmarks

#### 4.1.1 Myocardial alignment and sarcomere ultrastructure

In the native heart, cardiomyocytes are organized within a highly anisotropic myocardial microenvironment, in which coordinated cellular alignment is essential for efficient electromechanical coupling and force generation.^78^ A primary hallmark of structural maturity in cardiac MPS is the transition from disorganized, nascent bundles to more coordinated myofibrillar architectures.^79^, ^80^ Adult‐like cardiomyocytes exhibit a distinctive rod‐like cytoarchitecture, which is closely associated with efficient biomechanical force transmission and contractile performance.^81^, ^82^ This geometric and ultrastructural progression is not merely a morphological descriptor but also a useful benchmark for assessing the mechanofunctional status of engineered cardiac tissue models.^82^ Within the context of cardiac MPS, the fidelity of myocardial alignment and the hierarchical organization of sarcomeres therefore serve as fundamental metrics for structural validation.^79^, ^80^

Immature hiPSC‐CMs within early‐stage cardiac MPS often display disorganized, short, or incompletely assembled sarcomeric patterns.^82^ As cardiac MPS undergo maturation, these cells undergo progressive myofibrillar remodeling, leading to the formation of clearer and more periodic sarcomeric units with increasingly organized actin‐myosin arrays.^79^, ^80^ This maturation trajectory is commonly evaluated by the striated distribution of sarcomeric proteins, including cardiac troponin T (cTnT), together with maturation‐associated changes in myosin light chain isoform patterning.^79^, ^82^ Notably, resting sarcomere length serves as a critical quantitative benchmark. While early‐stage organoids or immature hiPSC‐CMs often remain at a fetal‐ or neonatal‐like phenotype with relatively short sarcomeres, advanced maturation strategies—such as topographical guidance, mechanical conditioning, or electrical stimulation—seek to approach the adult physiological range of approximately 2.2 μm.^82^, ^83^ For instance, the Biowire platform exemplifies how chronic electrical stimulation can enhance cellular alignment, myofibrillar organization, and structural maturation, thereby partially bridging the gap between fetal‐like in vitro tissues and more adult‐like functional phenotypes.^83^

At a finer subcellular scale, additional organizational features provide further evidence of advanced maturation. In particular, the emergence of well‐defined striated sarcomeres, including more ordered Z‐disc and A‐band organization, together with the localization of Cx43 at intercellular junctions, provides an important structural benchmark for evaluating the cardiac MPS's capacity to recapitulate higher‐order cardiac tissue behavior.^84^

#### 4.1.2 Chamber‐like morphogenesis in self‐organizing COs

Beyond cellular alignment and multicellular composition, chamber‐like morphogenesis and spatial compartmentalization constitute higher‐order structural features relevant to the maturation of self‐organizing COs. These features should be regarded as platform‐specific benchmarks rather than universal criteria for all cardiac MPS, because most engineered cardiac tissue models are not designed to reproduce developmental chamber morphogenesis. In contrast to the native heart, where anatomically discrete chambers and outflow structures are fully established, current self‐organizing COs more commonly recapitulate early morphogenetic events such as lumen formation, cavity development, and regional lineage patterning rather than fully mature chamber architecture. Temporal modulation of Wnt signaling has been shown to generate vascularized COs containing endothelial‐bounded chambers with improved electrophysiological properties relative to cardiomyocyte‐only organoids.^85^ In addition, retinoic acid (RA)‐dependent patterning strategies have enabled the generation of atrial‐like and ventricular‐like heart organoids under RA^+^ and RA^−^ conditions, respectively, supporting the use of COs for modeling chamber‐related lineage specification and chamber‐defect phenotypes.^4^ Evaluation of these spatial features increasingly relies on non‐destructive 3D imaging. X‐ray phase‐contrast tomography (XPCT) enables multiscale isotropic histological reconstruction of heart‐forming organoids at subcellular resolution, whereas optical coherence tomography (OCT) supports longitudinal visualization of cavity emergence, internal interconnections, and dynamic contraction patterns during organoid development.^86^, ^87^

#### 4.1.3 Multicellular architecture

The evolution of cardiac MPS from simple cardiomyocyte aggregates to sophisticated biomimetic systems is closely associated with increasingly stringent criteria for structural and functional maturation.^79^, ^88^ The presence and organized distribution of non‐myocyte lineages—including fibroblasts, endothelial cells, and pericyte‐containing vascular populations—now serve as important benchmarks for assessing tissue‐level maturity and physiological fidelity.^88^ Cardiac fibroblasts contribute to extracellular matrix deposition and remodeling, thereby helping establish a more native‐like microenvironment enriched in fibrillar collagens, fibronectin, and related matrix components. The spatial patterning and extent of these ECM features, quantifiable via high‐resolution imaging, reflect the cardiac MPS's structural integrity and its capacity to model fibrotic remodeling.^88^, ^89^ Furthermore, the inclusion of vascular‐supporting cells can promote selected aspects of cardiac tissue maturation, matrix remodeling, and contractile performance; in particular, paracrine platelet‐derived growth factor receptor β (PDGFRβ) signaling from vascular populations contributes to extracellular matrix remodeling that augments contractile performance.^88^ Beyond structural scaffolding, the recent incorporation of cardiac‐resident macrophages and sympathetic neurons introduces dynamic immuno‐cardiac and neuro‐cardiac regulation, thereby further elevating the physiological relevance of these models.^90^, ^91^ Consequently, the synergistic crosstalk between these diverse cell types and their responsiveness to biophysical cues, such as matrix stiffness, should be incorporated into a broader framework for assessing the maturity and physiological fidelity.^92^

#### 4.1.4 Molecular validation of developmental stage‐specific markers

Beyond morphological assessment, multidimensional molecular profiling of transcriptomic and proteomic states is increasingly required for rigorous evaluation of the developmental maturity and physiological fidelity of cardiac MPS.^79^, ^93^, ^94^ Contemporary evaluation paradigms have progressed beyond the simple detection of pan‐cardiac lineage markers toward the quantitative assessment of developmentally regulated molecular transitions.^92^, ^95^ Specifically, in cardiac MPS, the developmental transition from slow skeletal troponin I (TNNI1) to cardiac troponin I (TNNI3) is widely regarded as a maturation‐associated molecular signature consistent with progression toward a more advanced contractile phenotype; this process is often accompanied by broader myosin heavy‐chain and sarcomeric isoform remodeling rather than reliance on any single transcript ratio.^92^, ^95^ Notably, TNNI3 accounted for up to approximately 18% of the combined TNNI1 and TNNI3 signal, corresponding approximately to the molecular maturation level of the human fetal heart at 20 weeks of gestation.^79^ This finding also illustrates that substantial in vitro maturation does not necessarily indicate attainment of a fully adult molecular phenotype. The use of single‐cell RNA sequencing (scRNA‐seq) enables high‐resolution cellular atlasing and developmental trajectory analysis, thereby facilitating quantitative comparison between cardiac MPS transcriptional states and in vivo human cardiac references.^79^, ^93^ Furthermore, because transcript abundance does not always correspond directly to protein‐level output owing to post‐transcriptional and protein‐level regulation, orthogonal protein‐based and spatial validation remain indispensable.^94^ Accordingly, multiplexed immunofluorescence and related spatial profiling approaches can be used to map sarcomeric organization, ion channel distribution, and junctional complexes such as Cx43, thereby confirming whether molecular maturation is accompanied by the structural establishment of a functional electromechanical syncytium.^79^, ^80^

### 4.2 Functional maturation assessment

To provide physiologically and pharmacologically informative readouts, cardiac MPS must achieve a level of functional maturity appropriate to their intended application. Crucially, functional maturity is not an independent variable but the physiological manifestation of underlying structural refinement—a principle known as the structure–function interdependence. Accordingly, their evaluation requires a multi‐parametric framework that integrates electrophysiology, mechanocontractile dynamics, and metabolic flux, going far beyond simple contractility assessments.

#### 4.2.1 Electrophysiological properties and ion channel functionality

The maturation of cardiac MPS is characterized by a progressive transition of electrophysiological properties toward a more adult‐like myocardial phenotype. Key hallmarks include the development of a more hyperpolarized resting membrane potential, more mature action potential morphology, coordinated remodeling of major ion currents, and enhanced responsiveness to neurohumoral stimulation such as β‐adrenergic signaling.^80^, ^96^ In early‐intensity electromechanically conditioned human engineered cardiac tissues, a conduction velocity of 25.0 cm/s was reported, providing a study‐specific quantitative reference for enhanced tissue‐level electrical propagation.^80^

These electrophysiological features enhance the value of cardiac MPS for preclinical investigation. In this context, the integration of multiparametric optical imaging has substantially improved the throughput and informational depth of functional screening.^97^ Specifically, state‐of‐the‐art optical platforms enable the simultaneous recording of transmembrane action potentials and intracellular calcium transients, the two core functional components underlying E‐C coupling. By using orthogonal fluorescent reporters, such as FluoVolt for membrane voltage and Rhod‐2 for calcium dynamics, these systems reduce dependence on traditional low‐throughput patch‐clamp approaches and allow parallel assessment of electrical activity and calcium handling for mechanistic evaluation of drug‐induced arrhythmogenic risk.^97^ Because calcium transients reflect E‐C coupling more directly than electrophysiological maturation alone, their interpretation should be integrated with contractile readouts rather than considered in isolation.^96^, ^97^

#### 4.2.2 Mechanocontractile dynamics and E‐C coupling

E‐C coupling constitutes the core functional process by which cardiomyocytes convert electrical excitation into mechanical output. A rigorous assessment of E‐C coupling maturity therefore requires high‐resolution analysis of calcium transient dynamics. In mature cardiomyocytes, an organized T‐tubule network supports synchronous Ca^2+^ release by enabling nanoscale coupling between L‐type Ca^2+^ channels and ryanodine receptor 2 (RyR2) within dyads, thereby promoting efficient calcium‐induced calcium release, mature sarcoplasmic reticulum Ca^2+^ handling, and faster, more spatially uniform Ca^2+^ transients.^96^ By contrast, immature hiPSC‐CMs typically exhibit rudimentary or absent T‐tubular structures, resulting in impaired dyadic organization, slower and smaller Ca^2+^ transients, greater reliance on trans‐sarcolemmal Ca^2+^ flux, prolonged decay kinetics, and weaker mechanical output.^96^ Thus, calcium cycling maturity and force generation should be interpreted as closely related, but not identical, functional layers of E‐C coupling.

Functionally mature cardiac MPS are therefore expected to exhibit stronger contractile force and more stable rhythmicity. At the highest rate of progressive stretch conditioning, hiPSC‐derived engineered heart tissues achieved a specific twitch force of 11.28 mN/mm^2^, representing a 5.1‐fold increase relative to fixed‐length controls, together with increased cardiomyocyte size, improved cellular and myofibrillar alignment, enhanced sarcomere organization, and upregulation of an adult cardiomyocyte‐associated transcriptional program.^98^ To evaluate tissue‐level functional syncytia, advanced signal‐processing tools such as PhysioMEA can be applied to multi‐electrode array data.^99^ By quantifying beat‐rate variability and mapping the 2D dynamics of electrophysiological biomarkers, these platforms can identify regional conduction abnormalities and evaluate functional synchronization across the tissue.^99^ In more advanced engineered cardiac tissues, improved ultrastructural organization, polarized Cx43 localization, and enhanced electromechanical coupling have been observed, although the complete biogenesis of a mature and well‐organized T‐tubule network remains a major limitation in current cardiac MPS.^80^, ^96^

#### 4.2.3 Metabolic reprogramming and energetic homeostasis

Cardiomyocytes are highly energy‐demanding cells whose functional integrity relies on a sophisticated and efficient metabolic framework. Consequently, characterizing the metabolic profile of cardiac MPS is indispensable for benchmarking their physiological status and developmental maturity. Immature cardiomyocytes predominantly rely on anaerobic glycolysis, characterized by diminished mitochondrial oxidative phosphorylation and suboptimal ATP yield.^92^, ^100^ To quantify this metabolic state, extracellular flux analysis (e.g., Seahorse XF Assay) is employed to measure the Oxygen Consumption Rate (OCR) and Extracellular Acidification Rate (ECAR), where a low OCR/ECAR ratio serves as a hallmark of glycolytic immaturity.^100^

In contrast, mature cardiomyocytes undergo a profound metabolic reprogramming toward fatty acid β‐oxidation as their primary energy substrate. This transition is intrinsically coupled with a marked increase in mitochondrial biogenesis and the development of dense, complex mitochondrial cristae. In early‐stage, intensity‐trained, electromechanically conditioned hiPSC‐derived cardiac tissues, mitochondria occupied 30% of the analyzed area, a level comparable to that of adult human myocardium; this adult‐like ultrastructural feature was accompanied by increased oxygen consumption and a shift toward oxidative metabolism.^80^ Such ultrastructural refinement provides a robust energetic reservoir essential for sustaining high‐intensity mechanical contractility.^92^, ^101^

To overcome the metabolic immaturity of hiPSC‐CMs, several bioengineering strategies have been implemented. For instance, substrate switching—the depletion of glucose supplemented with physiological fatty acids (e.g., palmitate, oleate) and galactose—effectively forces a metabolic reprogramming toward oxidative states.^92^, ^101^ Furthermore, targeted interventions utilizing thyroid hormones or chronic high‐frequency electrical stimulation have been shown to synergistically promote mitochondrial maturation and enhance oxidative metabolic flux.^80^, ^102^

Taken together, the assessment of cardiac MPS maturation requires an integrated interpretation of structural organization, multicellular composition, electrophysiological behavior, E‐C coupling, and metabolic competence. No single metric can adequately define maturity in isolation. Rather, the translational value of cardiac MPS depends on whether these features converge toward a coherent adult‐like phenotype and whether functional performance is supported by an appropriate degree of underlying structural refinement.

### 4.3 Integrated summary of maturation metrics and analytical approaches

Because the maturation of cardiac MPS is multidimensional, no single biomarker, functional readout, or analytical method is sufficient to establish an adult‐like phenotype. Table 3 consolidates the principal structural, molecular, electrophysiological, calcium‐handling, contractile, and metabolic maturation metrics, together with representative analytical approaches and commonly used benchmark features. The listed benchmarks should be interpreted primarily as comparative reference points rather than universal acceptance thresholds, because quantitative measurements may vary substantially with cardiac subtype, culture medium, tissue geometry, assay platform, and normalization strategy.

## 5 APPLICATIONS OF CARDIAC MPS IN CARDIOVASCULAR RESEARCH

Cardiac MPS can help bridge the translational gap between traditional animal models and simplified 2D cell cultures. By recapitulating key features of the spatiotemporal cellular heterogeneity, electromechanical coupling, and metabolic signatures of the native myocardium, these 3D microphysiological systems have rapidly transitioned from proof‐of‐concept constructs to increasingly useful research tools. Their current applications can be broadly categorized into four major domains: modeling human cardiac development, interrogating disease mechanisms, supporting translational pharmacology, and enabling regenerative medicine‐oriented research and reparative tissue evaluation (Figure 4). Importantly, these application domains place distinct demands on platform architecture, multicellular complexity, and functional maturity: developmental studies prioritize morphogenetic fidelity, disease modeling requires pathology‐relevant multicellular states, pharmacology depends on reproducibility and quantifiable functional readouts, whereas regenerative applications additionally require structural robustness and integration potential.

**FIGURE 4:**
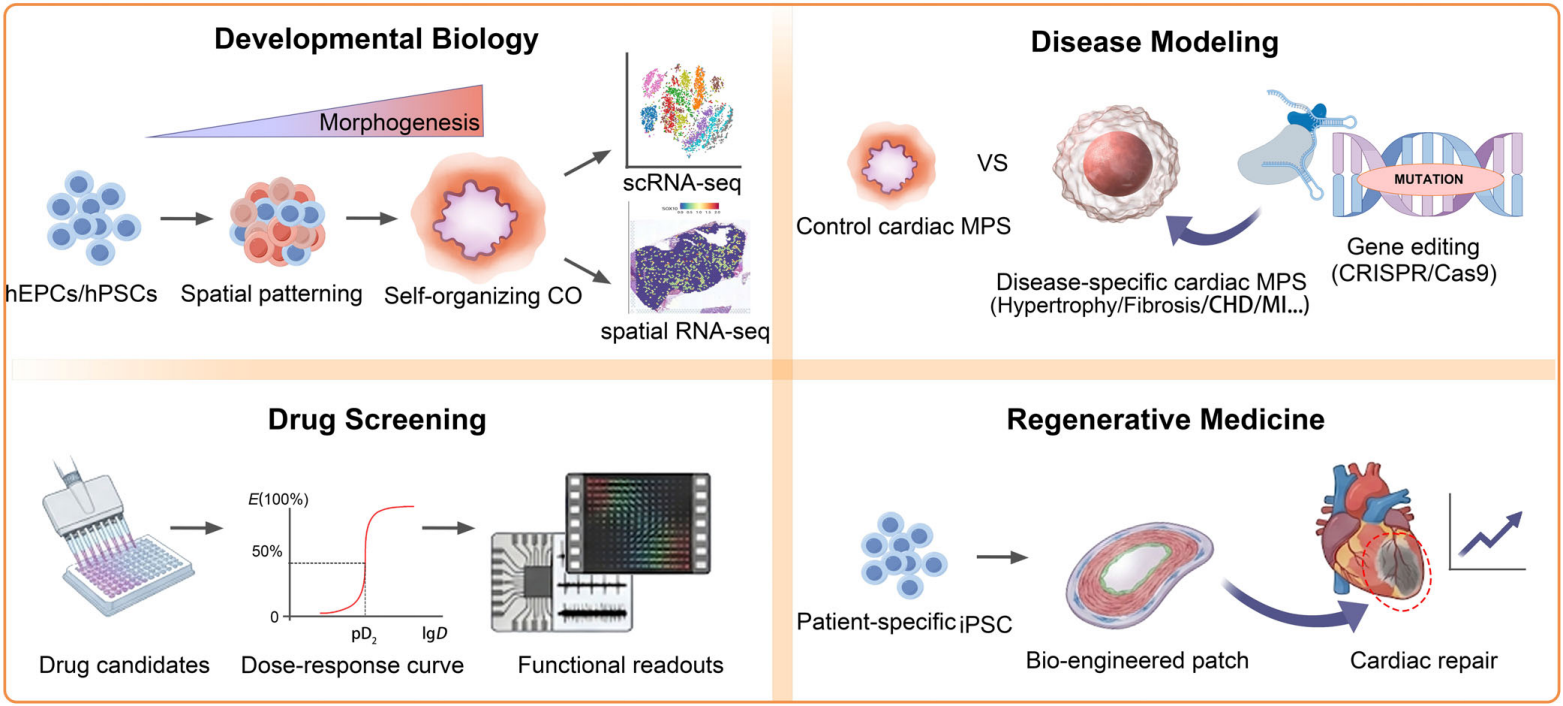
Major applications of cardiac MPS in cardiovascular research. This figure summarizes the primary applications of cardiac MPS in development biology, disease modeling, drug screening, and regenerative medicine.

### 5.1 Deciphering the mechanisms of cardiac development

The principal value of self‐organizing COs lies in their ability to capture lineage specification, spatial morphogenesis, and early tissue patterning within the same experimental system, thereby enabling the dissection of stage‐specific gene regulatory networks and morphogenetic checkpoints that are otherwise difficult to access in vivo.^4^ For example, self‐organizing cardioid models have revealed a context‐dependent regulatory relationship between HAND1 and NKX2‐5, in which heart and neural crest derivatives expressed 1 (HAND1) act upstream of NK2 homeobox 5 (NKX2‐5) in cardiac mesoderm, whereas NKX2‐5 becomes upstream of HAND1 at later cardiomyocyte stages; notably, HAND1 deficiency results in more severe morphogenetic defects, including chamber formation failure, compared to NKX2‐5 deficiency in these early chamber‐forming systems.^4^

Building upon these molecular insights, recent advances in organoid technology have enabled the recapitulation of increasingly complex architectural features of the embryonic heart. Heart‐forming organoids, for example, structurally mirror the early native heart anlagen prior to heart tube assembly.^103^ These models comprise organized myocardial layers, endothelial‐like linings, and septum transversum‐like structures, and they further incorporate spatially and molecularly distinct foregut endoderm and vascular networks.^103^ Furthermore, the integration of 2D and 3D differentiation protocols has enabled the efficient generation of progenitor subpopulations with distinct first heart field, anterior second heart field, and posterior second heart field identities, thereby facilitating the reconstruction of multiple embryonic cardiac compartments in vitro.^20^

This spatial and lineage‐specific control allows advanced multi‐chamber cardioids to reproduce the major embryonic cardiac compartments, including ventricular, atrial, outflow tract, and atrioventricular canal‐like domains, with compartment‐specific gene expression profiles and functional properties.^20^ In parallel, patterned primitive heart organoid models have enabled the generation of large atrial‐ and ventricular‐like chambers together with proepicardial organ formation and have further demonstrated retinoic acid‐mediated anterior–posterior patterning that resembles developmental events of the post‐heart tube primitive heart.^104^ Taken together, self‐organizing COs are particularly well suited to developmental biology because they allow signaling, lineage allocation, and tissue patterning to be interrogated in an integrated, human‐specific context rather than as isolated endpoints.

### 5.2 Disease modeling and pathogenic insights

The suitability of different cardiac MPS formats for disease modeling is highly disease‐ and context‐dependent. These platforms are particularly informative when disease mechanisms are governed by multicellular organization, developmental patterning, tissue‐level contractile behavior, or spatially structured microenvironments. Accordingly, self‐organizing COs are particularly informative for investigating early developmental processes and selected pathogenic mechanisms relevant to congenital heart defects; patient‐specific and gene‐edited hPSC‐derived cardiac models are useful for reproducing selected phenotypes of monogenic cardiomyopathies; and engineered cardiac tissue models with controlled oxygenation or perfusion are especially relevant to selected aspects of ischemic injury and myocardial infarction. By contrast, adult‐onset and chronic cardiovascular diseases remain more difficult to model comprehensively because many current hPSC‐derived cardiac systems retain fetal‐ or neonatal‐like electrophysiological, metabolic, and ultrastructural features, have limited functional vascularization and immune‐stromal complexity, and cannot fully reproduce systemic endocrine, immune, metabolic, renal, hepatic, vascular, and hemodynamic interactions.

#### 5.2.1 Modeling congenital heart defects

Congenital heart defects (CHDs), representing some of the most prevalent birth defects, involve complex interactions among genetic variation, environmental perturbation, and spatial morphogenetic abnormalities. Self‐organizing COs offer a uniquely tractable platform for dissecting these multifactorial pathogenic mechanisms. This is particularly important in CHDs because many defects reflect spatially coordinated developmental processes that cannot be adequately captured in conventional 2D systems. Building on the developmental capabilities described in Section 5.1, multi‐chamber self‐organizing COs can be used to investigate how perturbations in regional lineage allocation, chamber specification, and intercompartmental signaling generate CHD‐related phenotypes.^20^ Genetically, COs also provide a useful platform for modeling CHD‐associated molecular lesions. For instance, overexpression of microRNA‐187 in COs induces cardiac endothelial abnormalities that mirror clinically relevant CHD‐associated phenotypes, and these defects can be ameliorated by Nipped‐B homolog (NIPBL) supplementation.^105^

#### 5.2.2 Genetic cardiomyopathies

Inherited cardiomyopathies, primarily dilated cardiomyopathy (DCM) and hypertrophic cardiomyopathy, arise from a diverse genetic landscape. Patient‐specific and gene‐edited COs can help bridge the gap between genotype and pathological phenotype. Compared with simpler monolayer systems, they are particularly useful when mutation effects extend beyond cell‐autonomous dysfunction to altered calcium handling, multicellular stress responses, and tissue‐level contractile behavior. For DCM—characterized by ventricular dilation and systolic dysfunction—genome editing and patient‐specific hPSC‐derived models have enabled the interrogation of disease‐associated variants in a tissue‐relevant context. For example, frameshift variants in C10orf71 were identified as a cause of DCM, and gene‐edited COs recapitulated intrinsic contractile defects associated with these variants.^106^ The translational relevance of this finding was further supported in a C10orf71‐knockout mouse model, in which the cardiac myosin activator Omecamtiv mecarbil restored contractile function.^106^ This cross‐model validation underscores the role of organoids in disease‐gene discovery and highlights their synergy with in vivo models for therapeutic evaluation.

#### 5.2.3 Modeling ischemic injury and myocardial infarction

Myocardial infarction initiates a complex pathophysiological cascade, driving ischemic necrosis, sterile inflammation, and reactive fibrosis that ultimately culminate in maladaptive ventricular remodeling. To emulate this process in vitro, an engineered multicellular cardiac tissue model was developed with defined oxygen gradients, in some cases combined with neurohumoral stimulation such as noradrenaline. This strategy enables the reconstruction of infarct‐like, border‐zone‐like, and remote‐like regions within the same construct, thereby capturing the spatial heterogeneity that is a defining feature of post‐infarction myocardium.^107^ Furthermore, these models reproduce key hallmarks of myocardial infarction, including pathological metabolic remodeling, fibrosis, and altered calcium handling at transcriptomic and functional levels. Importantly, complementary engineered border‐zone models have further demonstrated that oxygen gradients themselves can induce region‐specific abnormalities in calcium cycling, contractile stress, and inflammatory signaling that are distinct from those observed under uniform hypoxic stress, reinforcing the concept that spatially heterogeneous oxygenation is a major determinant of post‐infarction tissue behavior.^108^ Consequently, such zonally compartmentalized platforms provide a relevant microenvironment for evaluating pro‐regenerative and anti‐fibrotic interventions, while more faithfully reflecting ischemic heterogeneity than conventional uniform hypoxia models.

### 5.3 Pharmacological applications

Cardiac MPS can recapitulate cardiac responses to pharmacological interventions and therefore provide a useful platform for therapeutic screening. These models are particularly valuable for evaluating candidate interventions in myocardial injury, fibrosis, and post‐infarction repair. For example, vascularized and chambered COs have been used to assess the protective effect of captopril after cryoinjury, showing reduced fibrotic remodeling together with improved contraction synchrony and calcium handling.^13^ More broadly, the value of cardiac MPS in therapeutic screening lies in their ability to integrate multicellular injury responses with measurable structural and functional readouts.

Drug‐induced cardiotoxicity, especially in cardio‐oncology, remains a major challenge in drug development. Because conventional 2D monocultures do not adequately capture tissue‐level and multicellular responses, cardiac MPS have emerged as more informative models for toxicity assessment. Multicellular COs have been used to model doxorubicin‐induced cardiotoxicity, reproducing structural injury, collagen accumulation, endothelial dysfunction, and impaired contractile performance within the same tissue‐like system.^89^ Beyond conventional drugs, organoid‐on‐a‐chip platforms have also been applied to environmental toxicology. For instance, cardiac organoid‐on‐a‐chip models have shown that polystyrene nanoplastics induce oxidative stress and mitochondrial dysfunction and aggravate myocardial injury‐related phenotypes.^109^ At the platform level, the integration of cardiac MPS‐based assays with automated imaging, microfluidic multiplexing, and AI‐assisted phenotypic analysis is expected to improve the throughput and consistency of pharmacological profiling.^110^

### 5.4 Regenerative medicine and cardiac repair

#### 5.4.1 Platforms for studying cardiac regeneration and repair mechanisms

Beyond disease modeling, cardiac MPS provide a human‐relevant platform for investigating why the injured adult heart undergoes incomplete repair rather than true regeneration. Their major advantage lies in enabling repair to be studied as a multicellular and spatially coordinated process, rather than as an isolated cardiomyocyte response. Recent epicardioid and multicellular cardiac tissue studies have shown that repair outcomes are shaped by interactions among the epicardium, epicardium‐derived stromal states, endothelial compartments, and myocardium, which together regulate myocardial support, fibroblast specification, extracellular matrix remodeling, and the balance between adaptive repair and maladaptive fibrosis.^19^, ^111^ Single‐cell analyses further indicate that the epicardium functions as an active signaling compartment, whereas multicellular repair models suggest that pathological remodeling depends not simply on fibroblast abundance, but on the emergence and persistence of specific fibroblast states under injury‐related stimulation.^19^, ^111^ In parallel, engineered multicellular heart repair models that reproduce hypoxic stress, fibrosis, and electrophysiological dysfunction in a staged manner permit evaluation of repair‐stage‐specific interventions rather than antifibrotic strategies only under end‐stage conditions.^111^ Collectively, these platforms shift the experimental focus from single‐cell survival toward the coordinated regulation of tissue repair programs.

#### 5.4.2 Engineering cardiac tissue models for reparative tissue construction and translational evaluation

A more translational application of engineered cardiac tissue models lies in reparative tissue construction. Here, the goal is not merely to model repair, but to generate cardiac tissues with sufficient structural stability, vascular support, and electromechanical competence to contribute to myocardial recovery. This objective is becoming increasingly plausible through the integration of perfusable extracellular microenvironments, biomaterial‐guided organization, and scalable fabrication strategies.^112^, ^113^, ^114^ Perfusable engineered human cardiac tissues established within heart extracellular matrix‐based microenvironments have shown improved maturation and utility across disease modeling, drug testing, and regenerative applications.^112^ At a smaller modular scale, cryopreserved 3D microcardiac spheroids have also shown feasibility for myocardial infarction therapy in vivo, supporting the practical value of storable and administrable tissue units.^113^ More importantly, engineered heart muscle allografts have recently demonstrated durable remuscularization and functional wall augmentation in rhesus macaques, representing a substantial step toward preclinical cardiac repair.^114^

However, the current bottleneck is not simply initial graft survival after implantation, but the achievement of long‐term graft persistence and stable vascular, mechanical, and electrical integration with the host. Large‐animal studies indicate that post‐transplantation arrhythmias remain a major concern, highlighting that structural engraftment alone does not guarantee safe electrical integration.^114^, ^115^ More broadly, this risk likely reflects both incomplete graft‐host electrical coupling and insufficient control over the intrinsic electrophysiological properties of implanted tissues before transplantation.^114^, ^115^ In addition, immune compatibility, perfusion scaling, reproducible manufacturing at clinically relevant dimensions, and long‐term safety remain unresolved.^112^, ^114^ Accordingly, the near‐term value of engineered cardiac tissue models may lie less in immediate myocardial replacement than in serving as an intermediate translational platform linking human‐specific repair biology with biofabrication‐based therapeutic testing.

## 6 CHALLENGES AND FUTURE PERSPECTIVES

Although cardiac MPS have emerged as promising platforms for modeling cardiac development and disease, evaluating drug responses, and exploring regenerative strategies, their broader experimental and translational utility remains constrained by several unresolved technical limitations. At present, the major challenge for the field is not simply to generate increasingly complex tissue architectures and platform configurations, but to develop cardiac MPS that are physiologically relevant, experimentally reproducible, and suitable for defined translational applications. In this context, future progress will depend on coordinated advances in tissue maturation, long‐term stability, systemic relevance, manufacturing consistency, and data‐guided cultivation control (Figure 5).

**FIGURE 5:**
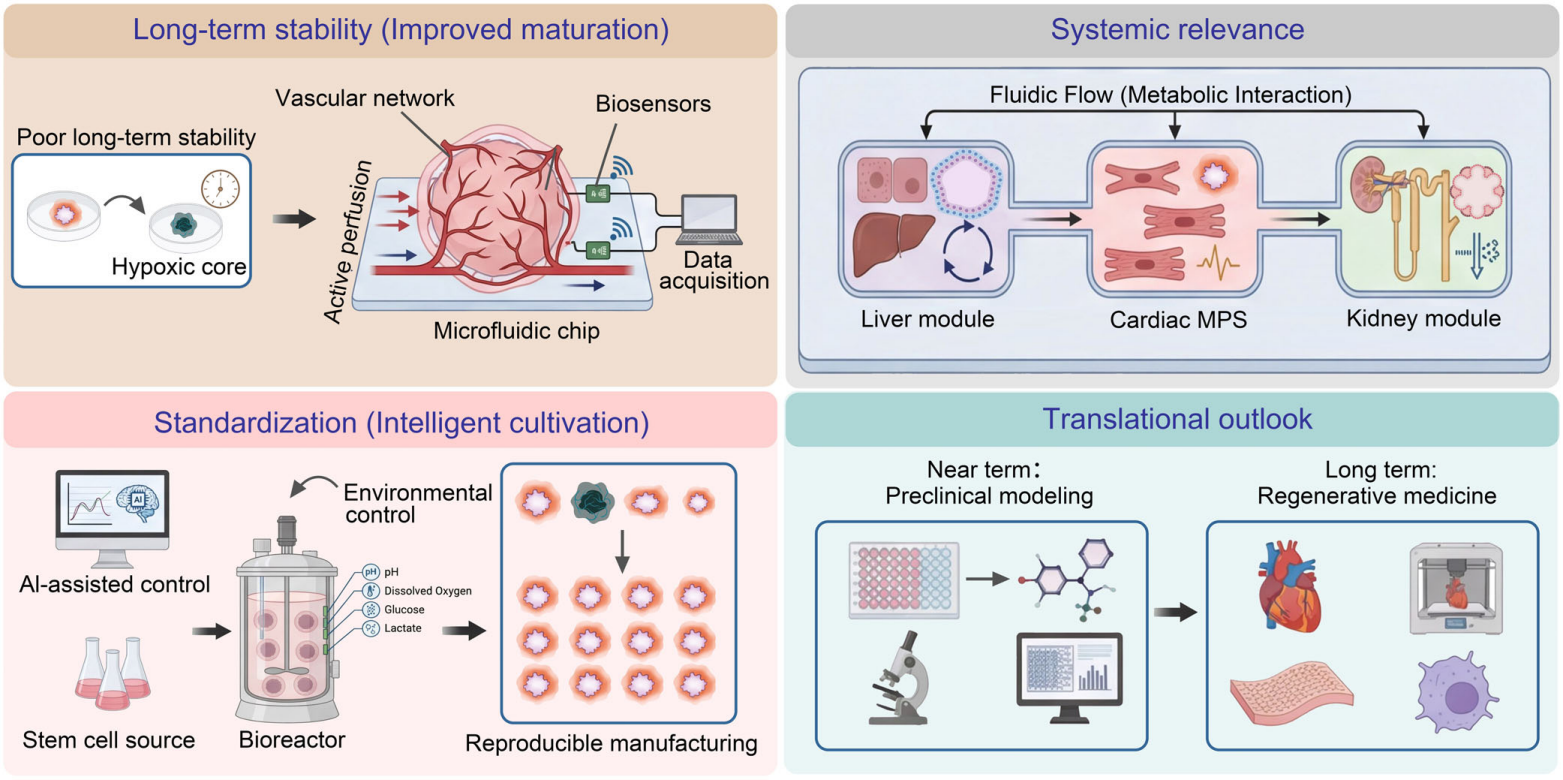
Challenges and future perspectives in advancing cardiac MPS. This schematic summarizes the major directions for advancing cardiac MPS, with emphasis on long‐term stability and maturation, systemic relevance, cultivation standardization, and translational development. Together, these considerations define the key priorities for improving the physiological fidelity, reproducibility, and broader utility of cardiac MPS in cardiovascular research.

### 6.1 Vascularization, maturation, and long‐term stability

A central limitation of current cardiac MPS is the incomplete establishment of vascularized, adult‐like cardiac tissue. In most systems, oxygen and nutrient delivery, along with waste removal, rely predominantly on passive diffusion, as a functional, perfusable vascular network has not yet been achieved. Once organoid size exceeds the diffusion range, the inner region becomes vulnerable to hypoxia, nutrient deprivation, and secondary injury, limiting tissue thickness, viability, and complexity.^85^, ^116^ Although endothelial co‐differentiation, co‐culture, and pro‐angiogenic patterning have enabled the formation of vascular‐like cardiac MPS, these approaches have not yet achieved a mature, hierarchically branched, and truly perfusable vascular architecture: a key requirement for mature cardiac functionality.^85^, ^117^, ^118^

This vascular limitation is closely linked to the broader problem of incomplete maturation and poor long‐term phenotypic stability.^118^, ^119^ Most hPSC‐derived cardiac MPS still resemble fetal‐ or neonatal‐like myocardium rather than fully recapitulating the electromechanical, metabolic, and ultrastructural characteristics of the adult human heart. In prolonged culture, the stable maintenance of progressively mature phenotypes remains difficult, owing not only to inadequate vascular support but also to suboptimal culture media, extracellular matrix remodeling, and insufficient long‐term biophysical conditioning. As a result, current cardiac MPS remain less effective for modeling chronic cardiovascular disease, delayed pharmacodynamic responses, and cumulative cardiotoxicity associated with long‐term drug exposure.^79^, ^120^ However, the required degree and type of maturation should be defined according to the intended application rather than by a universal adult‐like endpoint.

Several emerging technologies may help address these limitations. Microfluidic platforms provide tighter control over fluid flow, shear stress, morphogen gradients, oxygen delivery, and mechanical stimulation than conventional static culture, thereby offering a more controllable microenvironment for both self‐organizing COs and engineered cardiac tissue models.^121^ Integration with biosensors may further enable non‐invasive and real‐time assessment of parameters such as contractility, electrophysiological activity, metabolic state, oxygen tension, and pH, which could improve longitudinal monitoring of cardiac MPS maturation and functional status.^122^ However, these advances should be regarded as enabling tools rather than definitive solutions, since their practical value will depend on whether they can produce sustained gains in vascular competence, adult‐like maturation, and long‐term phenotypic stability.

### 6.2 Systemic relevance and multi‐organ integration

A major limitation of current cardiac MPS is their inability to recapitulate systemic physiology. In vivo, cardiac function is integrated with other organ systems, such as the liver, kidneys, and vasculature, through metabolic, endocrine, and hemodynamic regulation. Therefore, many cardiovascular diseases and drug responses cannot be fully modeled in heart‐only systems.^123^ This limitation is particularly important in the evaluation of systemic toxicity, metabolism‐dependent cardiotoxicity, and human‐specific pharmacological responses that depend on extra‐cardiac organ function.^123^, ^124^

To address this gap, multi‐organ‐on‐chip systems are beginning to incorporate cardiac tissue alongside metabolically active modules, such as liver and kidney compartments, to better capture inter‐organ interactions and post‐metabolic drug effects. However, achieving functional maturity across all modules remains a significant challenge. These platforms represent an important direction for improving the systemic relevance of cardiac MPS‐based studies, but substantial technical barriers remain. Physiologically meaningful inter‐organ systems are difficult to establish because different organ modules often require incompatible culture conditions, fluidic scaling and media recirculation remain complex, and sufficient functional maturity must be maintained across all compartments simultaneously.^123^ Therefore, while multi‐organ integration is likely to become increasingly important for translational pharmacology and disease modeling, its utility will remain limited unless the cardiac component itself can achieve robust maturation and stable function. Moreover, most multi‐organ platforms remain at an early stage of development, and their predictive value has not yet been broadly validated across laboratories.

### 6.3 Intelligent cultivation, scalable manufacturing, standardization, and quality control

The future value of cardiac MPS depends on their scalability, reproducibility, standardization, and quality control. Current workflows show significant variability due to differences in cell lines, differentiation efficiency, operator handling, and culture format inconsistencies.^125^ Automated culture platforms and suspension bioreactors may improve process control and support more reproducible generation of self‐organizing COs, while stirred‐bioreactor systems may facilitate large‐scale production and monitoring of hPSC‐derived cardiomyocytes for subsequent use in engineered cardiac tissue models.^125^ Advanced biosensors and computational analysis may further enable continuous assessment of developmental state, functional maturation, and process consistency.^126^ Looking further ahead, AI‐assisted closed‐loop cultivation may enable a shift from empirically fixed protocols toward adaptive optimization strategies in which imaging, electrophysiological, and biochemical readouts are used to dynamically adjust culture conditions. However, these approaches remain at an early stage and require broader validation across laboratories, cell lines, and platform configurations. Such approaches may improve fabrication quality control, reduce inter‐batch variability, and enhance the robustness of cardiac MPS for disease modeling and drug screening.^127^

At the same time, the field will require more rigorous standardization of manufacturing and culture workflows, benchmarking strategies, and quality‐control criteria. Current efforts increasingly emphasize input‐cell qualification, control of aggregate size and differentiation efficiency, in‐process monitoring, and final‐product characterization. Future progress will depend on broadly accepted operating procedures covering stem cell sources, differentiation regimens, culture conditions, matrix specifications, and quality‐control metrics, particularly those related to cellular composition, structural organization, electrophysiology, contractility, calcium handling, and metabolic maturation.126 Benchmarking against stage‐matched human cardiac tissues, standardized reference datasets, and well‐characterized positive and negative control compounds may further improve cross‐platform and cross‐laboratory comparability.^125^ Importantly, improvements in production efficiency alone should not be interpreted as evidence of improved biological fidelity. The central goal is not merely to increase throughput, but to produce cardiac MPS that are sufficiently consistent, interpretable, and comparable across laboratories to support meaningful biological conclusions and downstream translational use.

### 6.4 Translational outlook

Looking ahead, the translational development of cardiac MPS is likely to proceed in two directions: more reliable preclinical modeling, which is achievable in the near term, and regenerative medicine applications, which remain more challenging due to the need for structural and functional maturity. In the near term, cardiac MPS may provide their greatest value as human‐relevant platforms for disease modeling, safety pharmacology, and drug‐response testing, especially with improved maturation and standardized manufacturing. In contrast, clinical translation in regenerative medicine remains more demanding, as it requires not only structural and functional tissue maturity, but also adequate vascular support, effective graft‐host electromechanical integration, favorable biomaterial performance, and immune compatibility. Importantly, the relative priority of these barriers is application‐ and stage‐dependent. For disease modeling and drug‐response testing, reproducibility, application‐appropriate maturation, and standardized validation may be more immediate priorities than further increases in structural complexity. For regenerative cardiac patches, perfusable vascularization is an upstream prerequisite for graft survival and scale‐up; once durable engraftment has been achieved, stable graft‐host electrical integration and control of post‐transplant arrhythmias are likely to become the more immediate safety concerns.

Despite these opportunities, several technical barriers continue to limit the translational use of cardiac MPS. These include incomplete or application‐mismatched maturation, limited development of mature, hierarchical, and sustainably perfusable vascular networks, insufficient long‐term phenotypic stability, donor‐ and iPSC line‐dependent differences in differentiation efficiency and cellular composition, matrix‐dependent batch variability, and limited representation of systemic inter‐organ interactions. For drug testing, the predictive value of standalone cardiac MPS remains constrained by the absence of hepatic metabolism, renal clearance, immune‐endocrine regulation, and systemic vascular hemodynamics, as well as by insufficient validation using standardized reference compounds. For regenerative applications, implantable engineered cardiac tissue models face additional challenges, including scalable manufacturing, long‐term graft survival and persistence, immune compatibility, graft‐host vascular and electrical integration, arrhythmogenic risk, long‐term safety, and regulatory qualification. Therefore, future progress should be assessed not only by increasing structural complexity but also by measurable improvements in maturation, vascular competence, reproducibility, assay predictiveness, and application‐specific validation.

Additional engineering approaches, including synthetic biology, programmable morphogen regulation, advanced biomaterials, and high‐resolution 3D bioprinting, may further improve control over tissue polarity, regional identity, and chamber‐like organization. Programmable morphogen regulation may enhance spatial patterning in self‐organizing COs, whereas advanced biomaterials and 3D bioprinting may provide greater control over the composition and architecture of engineered cardiac tissue models. The value of these strategies should ultimately be judged by whether they produce measurable improvements in functional performance, long‐term stability, reproducibility, and translational relevance. Overall, the field is moving beyond proof‐of‐concept model generation toward robust, interpretable, and application‐specific cardiac MPS, for which biological performance and validation are prioritized over maximal structural sophistication.

## 7 CONCLUSION

In conclusion, cardiac MPS have rapidly evolved from relatively simple self‐organizing constructs to increasingly sophisticated platforms that integrate developmental self‐organization with engineered cardiac tissue models. This progression has established cardiac MPS as highly promising models for cardiovascular research, enabling the exploration of cardiac development, disease mechanisms, pharmacological responses, and regenerative strategies. However, their value lies not merely in increasing structural complexity, but in achieving an application‐appropriate balance among multicellular organization, functional maturation, biological fidelity, and experimental reproducibility. Despite ongoing challenges related to vascularization, maturation, long‐term stability, and standardization, advancements in bioengineering, multi‐omics, and automated cultivation are steadily improving the fidelity and applicability of these models. Therefore, cardiac MPS should be regarded as an essential platform offering profound insights and substantial potential for applications in cardiovascular research.

**Abbreviations**
BMP4: bone morphogenetic protein 4
C10orf71: chromosome 10 open reading frame 71
COs: cardiac organoids
CHDs: congenital heart defects
cTnT: cardiac troponin T
CVDs: cardiovascular diseases
Cx43: connexin‐43
DCM: dilated cardiomyopathy
E‐C: excitation‐contraction
ECM: extracellular matrix
FGF2: fibroblast growth factor 2
HAND1: heart and neural crest derivatives expressed 1
hiPSC‐CMs: human induced pluripotent stem cell‐derived cardiomyocytes
hPSCs: human pluripotent stem cells
iPSCs: induced pluripotent stem cells
MPS: microphysiological systems
NKX2‐5: NK2 homeobox 5
OCT: optical coherence tomography
PDGFR‐β: platelet‐derived growth factor receptor beta
RA: retinoic acid
RyR2: ryanodine receptor 2
scRNA‐seq: single‐cell RNA sequencing
TNNI1: troponin I1
TNNI3: troponin I3
VEGF: vascular endothelial growth factor
WNT: wingless‐related integration site
XPCT: X‐ray phase‐contrast tomography

## AUTHOR CONTRIBUTIONS

Xinrui Wang conceptualized and designed the review. Rongfang Xie drafted the manuscript and prepared the figures. Haiyang Zhao revised the manuscript and compiled the tables. Yuqing Lei revised the manuscript. All authors contributed to refining the arguments and presentation, and all authors reviewed and approved the final version of the manuscript.

## FUNDING INFORMATION

This study was supported by the Fujian Provincial Natural Science Foundation of China (No. 2024J09048) and the Joint Funds for the Innovation of Science and Technology, Fujian province (No. 2021Y9185).

## CONFLICT OF INTEREST STATEMENT

The authors declare no conflict of interest.

## Data Availability

Data sharing not applicable to this article as no datasets were generated or analyzed during the current study.
